# Supplementary material for: USMB-shMincle: a virus-free gene therapy for blocking M1/M2 polarization of tumor-associated macrophages
Source: Mol Ther Oncolytics. 2021 Aug 25;23:26–37. doi: 10.1016/j.omto.2021.08.010 (PMC8463747; doi:10.1016/j.omto.2021.08.010)
Supplement: Document 1. Tables S1 and S2 and Figures S1–S10 [file mmc1.pdf]

OMTO, Volume *23*

Supplemental information

USMB-shMincle: a virus-free gene therapy for blocking M1/M2 polarization of tumor-associated macrophages

Vivian Weiwen Xue, Jeff Yat-Fai Chung, Philip Chiu-Tsun Tang, Alex Siu-Wing Chan, Travis Hoi-Wai To, Justin Shing-Yin Chung, Francis Mussal, Eric W.-F. Lam, Chunjie Li, Ka-Fai To, Kam-Tong Leung, Hui-Yao Lan, and Patrick Ming-Kuen Tang

Supplementary Table S1. Target sequences of the siRNA candidates.

**siRNA**	**Target sequence**
siMincle #1	GTCAGTGGCAATGGGTGGA
siMincle #2	GTTTCATCACCAGATGTGT
siMincle #3	CAGGTTCAGTCAAGAATTG

Supplementary Table S2. List of primers used in this study.

Mouse gene	Forward primer (5' to 3')	Reverse primer (5' to 3')
Gapdh	GCATGGCCTTCCGTGTTC	GATGTCATCATACTTGGCAGGTTT
Syk	CTACTACAAGGCCCAGACC	TGATGCATTCGGGGGCGTAC
P65	CGAATCCAGACCAACAATAACC	ACAGCAAGAAGATCTCATCCC
Cxcr4	TCCTCCTGACTATACCTGACTTCATCT	CCTGTCATCCCCCTGACTGAT

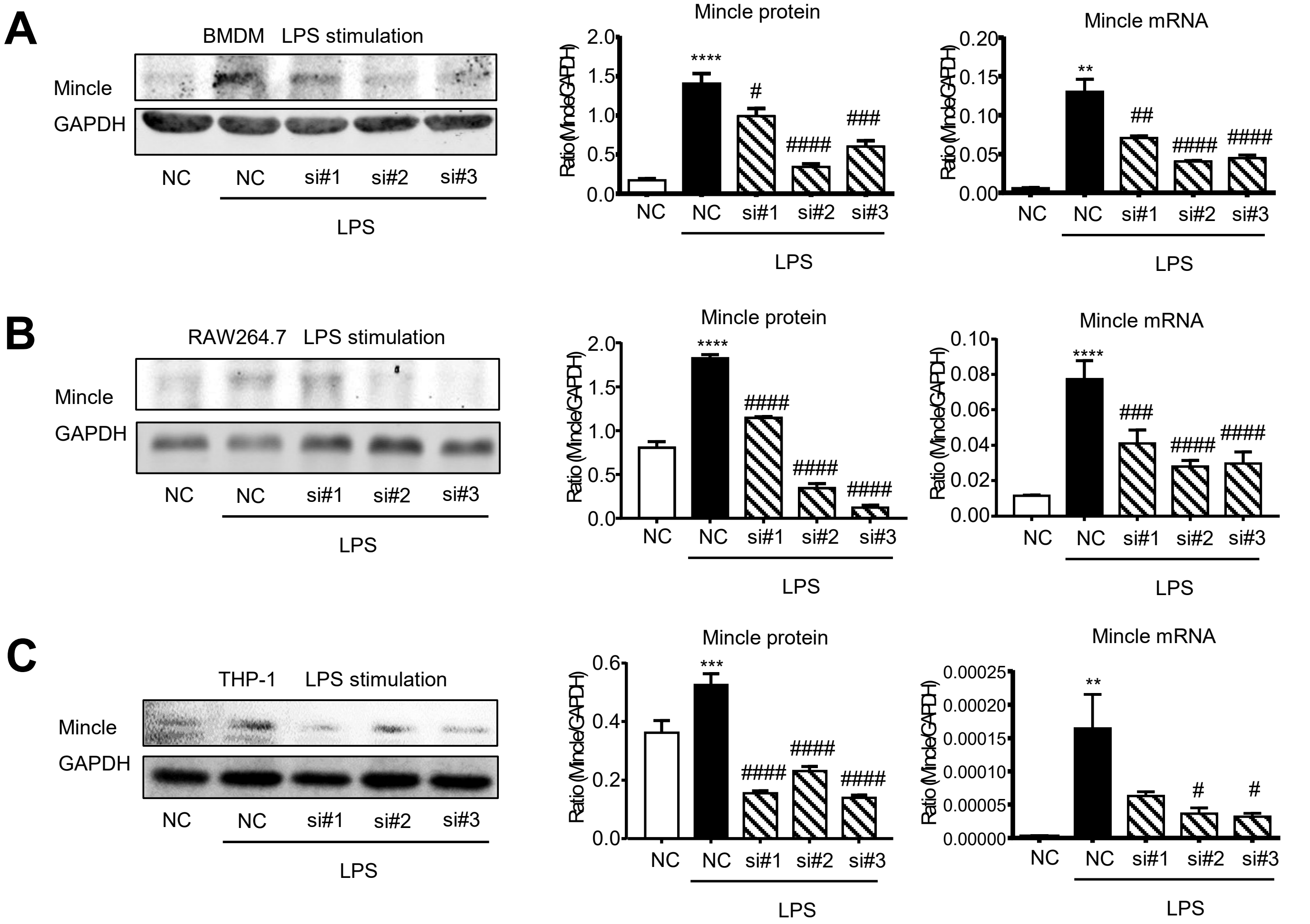

Figure S1. Screening of siRNA sequence for targeting both human and mouse Mincle *in vitro*. The knockdown efficiency of 3 siRNA sequences were tested on both murine and human macrophages in vitro. The siMincle #3 showed the best effect on Mincle suppression in mouse **(A)** BMDM and **(B)** RAW264.7 cells as well as human **(C)** THP-1 derived macrophages under LPS stimulation at 24h showing by Western blot and their quantification. Data represent mean ± SEM of 3 independent in vitro experiments. Statistical analysis based on ordinary one-way ANOVA, **** $p < 0.0001$, *** $p < 0.001$, ** $p < 0.01$ vs. NC; #### $p < 0.0001$, ### $p < 0.001$, ## $p < 0.01$, # $p < 0.05$ vs. NC+LPS.

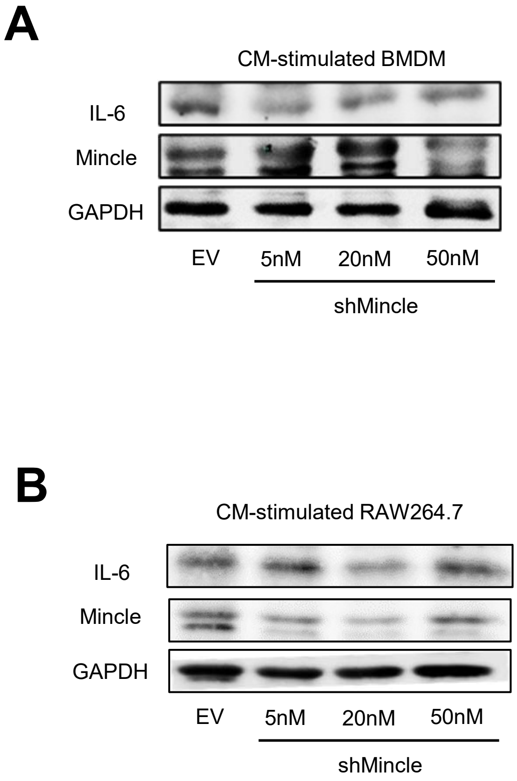

Figure S2. The shMincle effectively inhibits Mincle signaling *in vitro*. Western analysis confirmed that shMincle effectively inhibited the expression of Mincle and downstream effector IL-6 in **(A)** BMDM and **(B)** RAW264.7 cells under CM stimulation at 24h in vitro.

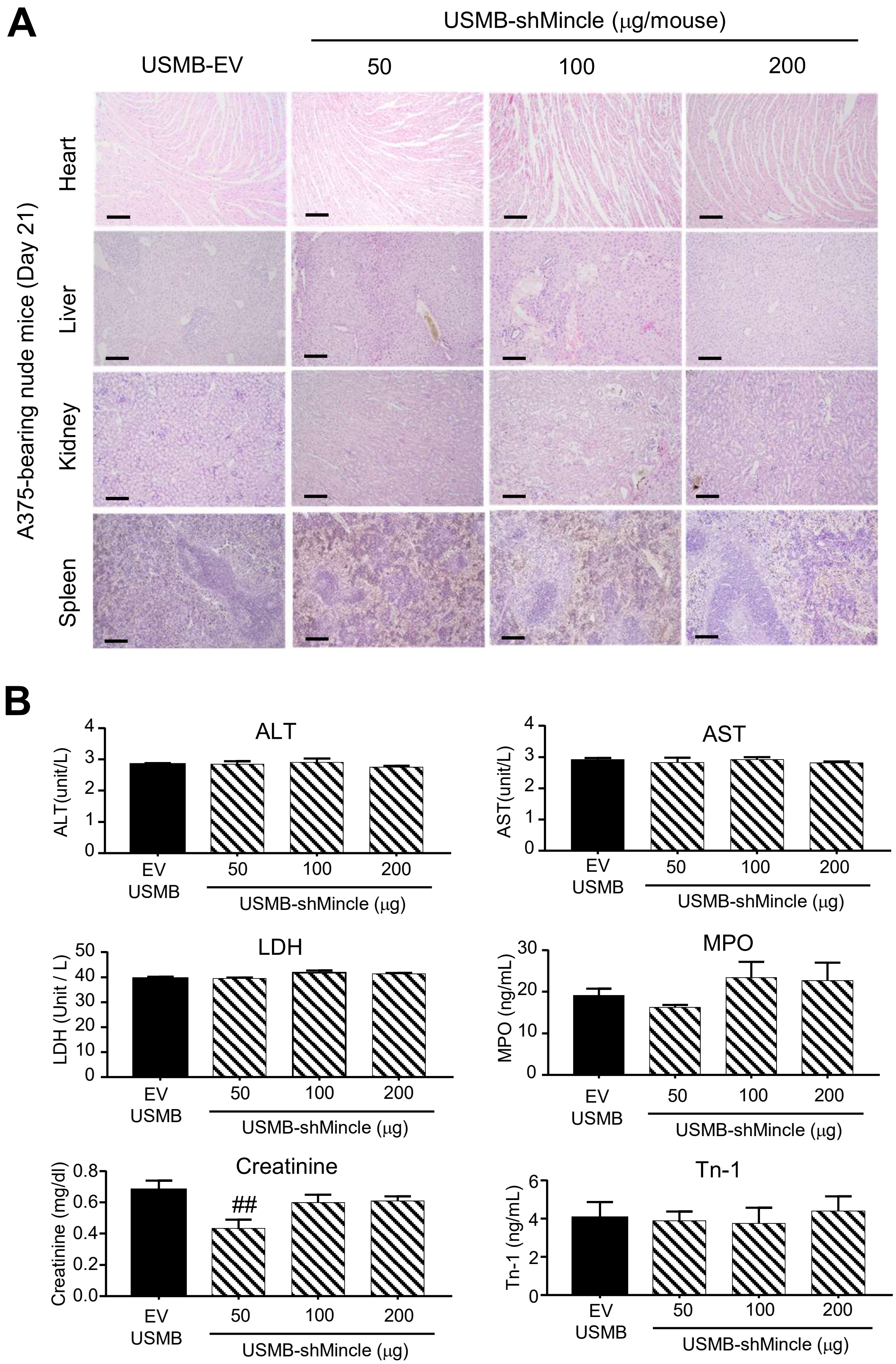

Figure S3. The safety of USMB-shMincle in human melanoma xenograft model A375. (A) H&E staining of important organs and **(B)** ELISA of the serum levels of ALT, AST, LDH, MPO, creatinine, and Tn-1 from the A375-bearing mice detected. Data represent mean ± SEM of 5 mice/group.

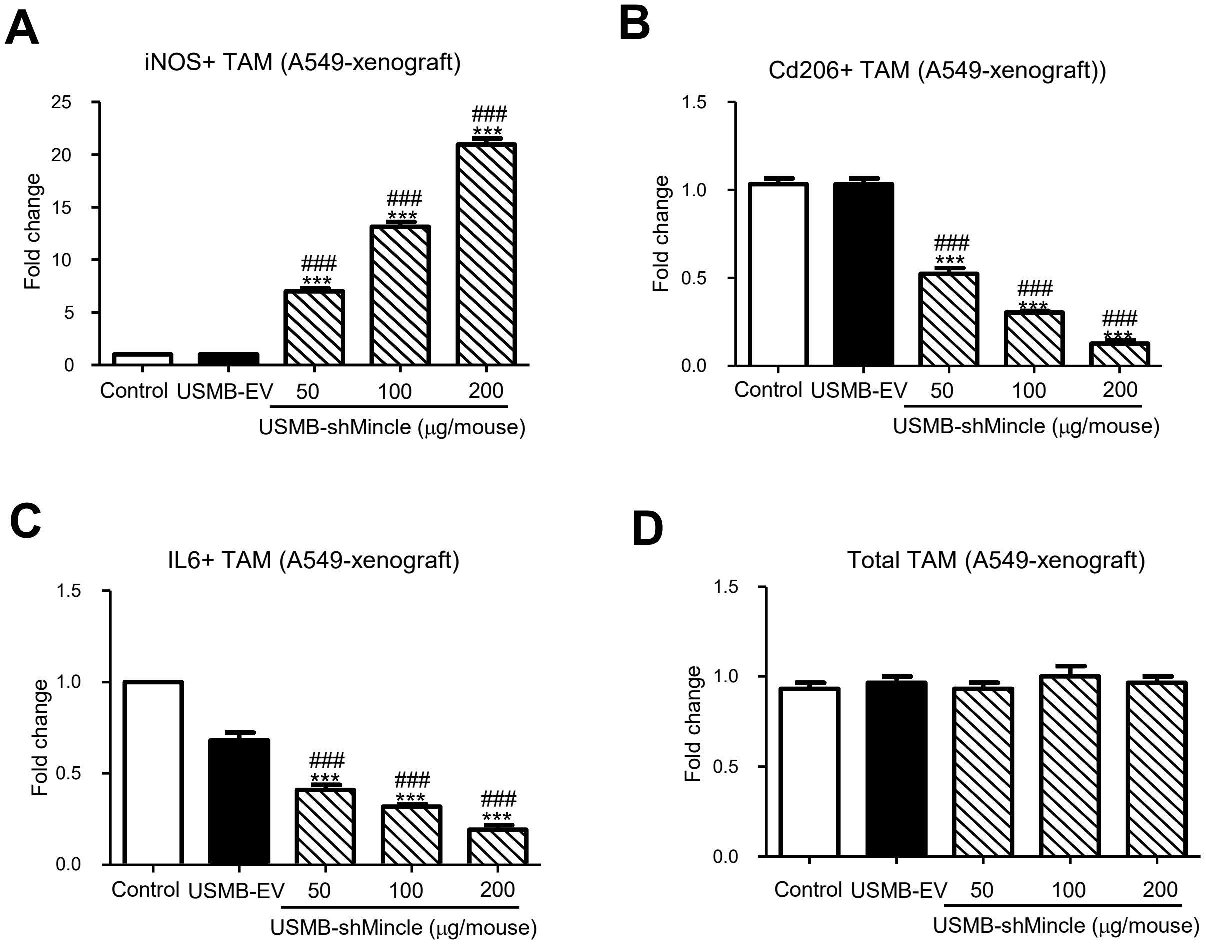

Figure S4. USMB-shMincle significantly altered the phenotype and function but not the number of TAM in the A549 xenografts *in vivo*. (A, B) The phenotypes of M1 (iNOS) and M2 (Cd206) and **(C)** expression of protumoral effector IL6 were dose-dependently changed in the USMB-shMincle compared to the baseline macrophage level of untreated control group, representing by the fold-change vs control group. **(D)** Interestingly, no significant change was detected in the number of TAMs among all groups by quantifying immunofluorescence result of Figure 6A. Data represent mean ± SEM of 5 mice/group. Statistical analysis based on ordinary one-way ANOVA.

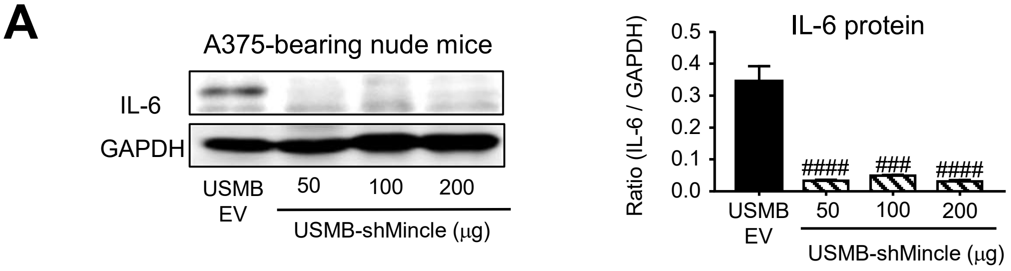

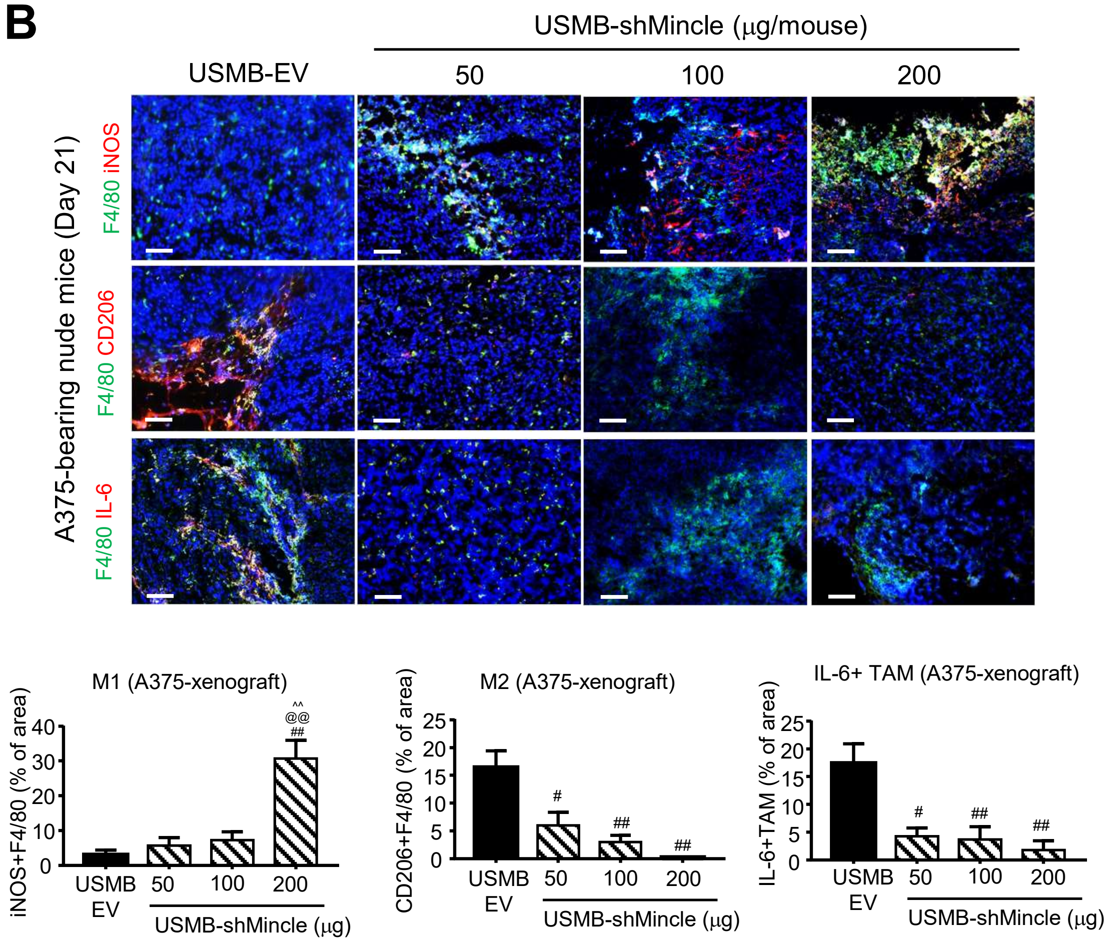

Figure S5. USMB-shMincle markedly inhibits M1/M2 transition in A375-bearing mice. **(A)** Treatment with USMB-shMincle markedly repressed the production of IL-6 in A375-xenografts showing by Western blot. **(B)** The USMB-shMincle largely enhanced the M1 phenotype iNOS but dramatically reduced the M2 phenotype CD206 and protumoral effector IL-6 in TAMs *in vivo*. Data represent mean ± SEM of 5 mice/group. Statistical analysis based on ordinary one-way ANOVA, #### $p < 0.0001$, ### $p < 0.001$, ## $p < 0.01$, # $p < 0.05$ vs. EV; @@ $p < 0.01$ vs. 50μg USMB-shMincle; ^^ $p < 0.01$ vs. 100μg USMB-shMincle.

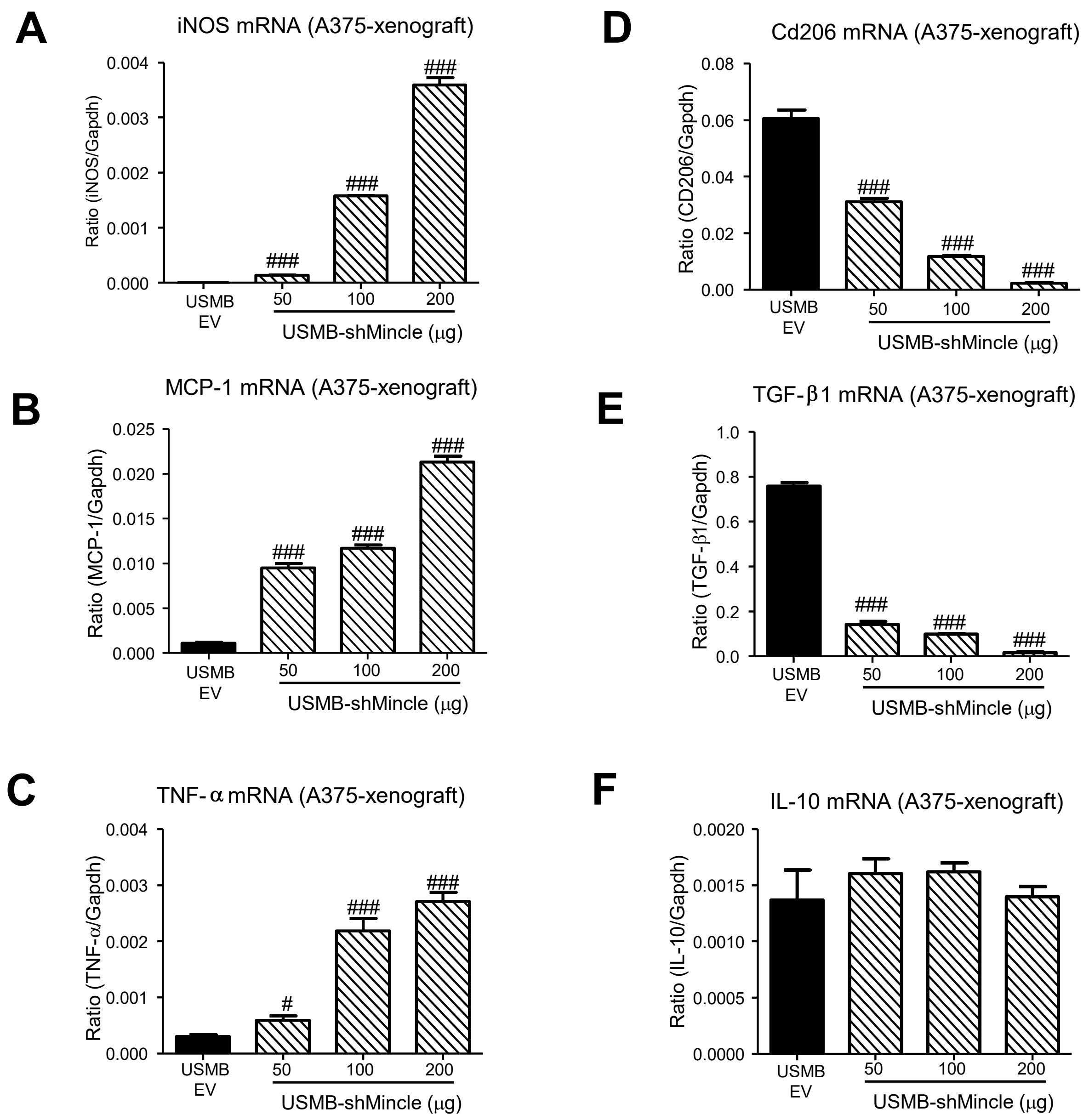

Figure S6. USMB-shMincle effectively enhances M1 function in A375-xenograft *in vivo*. Real-time PCR analysis showed that USMB-shMincle dose-dependently enhanced expressions of marker and effectors of **(A-C)** M1 (iNOS, MCP-1 and TNF-α), **(D-F)** but relatively reduction or insignificant change of M2 (Cd206, TGF-β1, IL-10) in the treated A375-xenografts. Data represent mean ± SEM of 5 mice/group. Statistical analysis based on ordinary one-way ANOVA, ### $p < 0.001$, # $p < 0.05$ vs. empty vector control (USMB-EV).

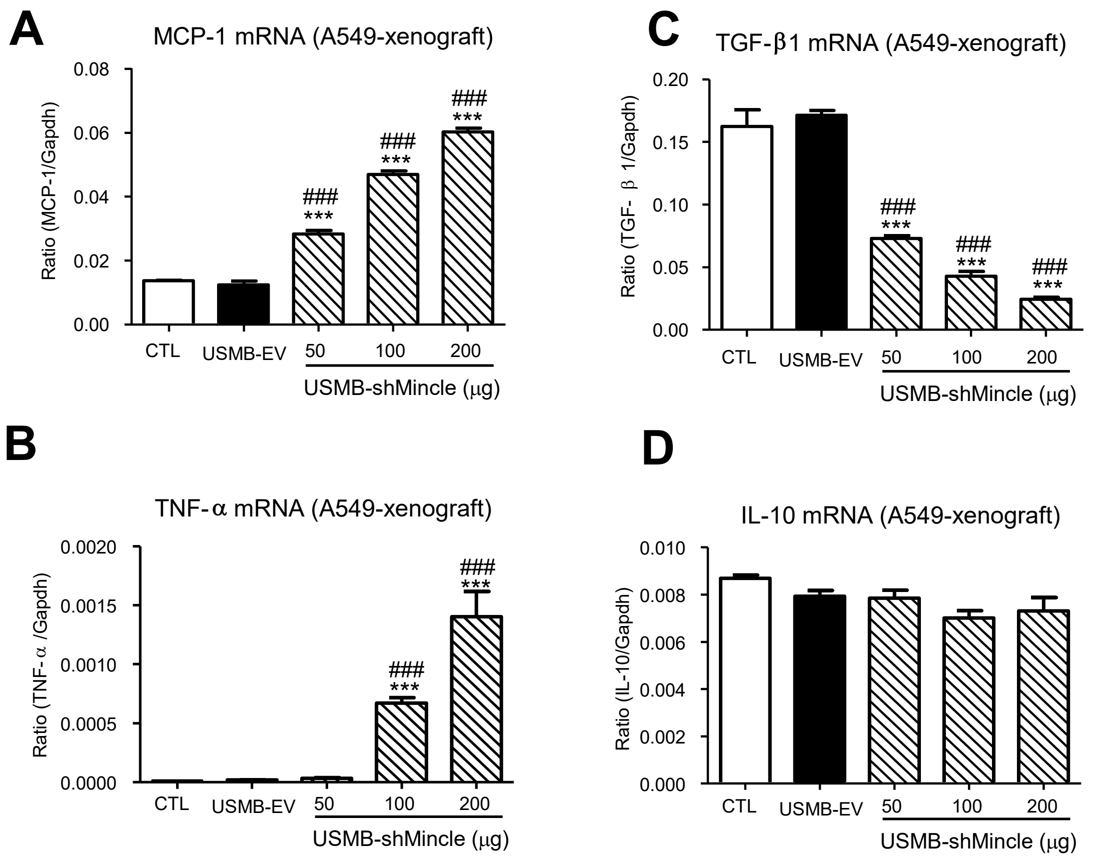

Figure S7. USMB-shMincle effectively enhances M1 function in A549-xenograft *in vivo*. Real-time PCR analysis showed that USMB-shMincle dose-dependently enhanced expressions of **(A-B)** M1 effectors MCP-1, TNF-α, **(C-D)** but relatively mild reduction or insignificant change of M2 effectors TGF-β1, IL-10 in the treated A549-xenografts in vivo. Data represent mean ± SEM of 5 mice/group. Statistical analysis based on ordinary one-way ANOVA, *** $p < 0.001$ vs. untreated control (CTL); ### $p < 0.001$ vs. empty vector control (USMB-EV).

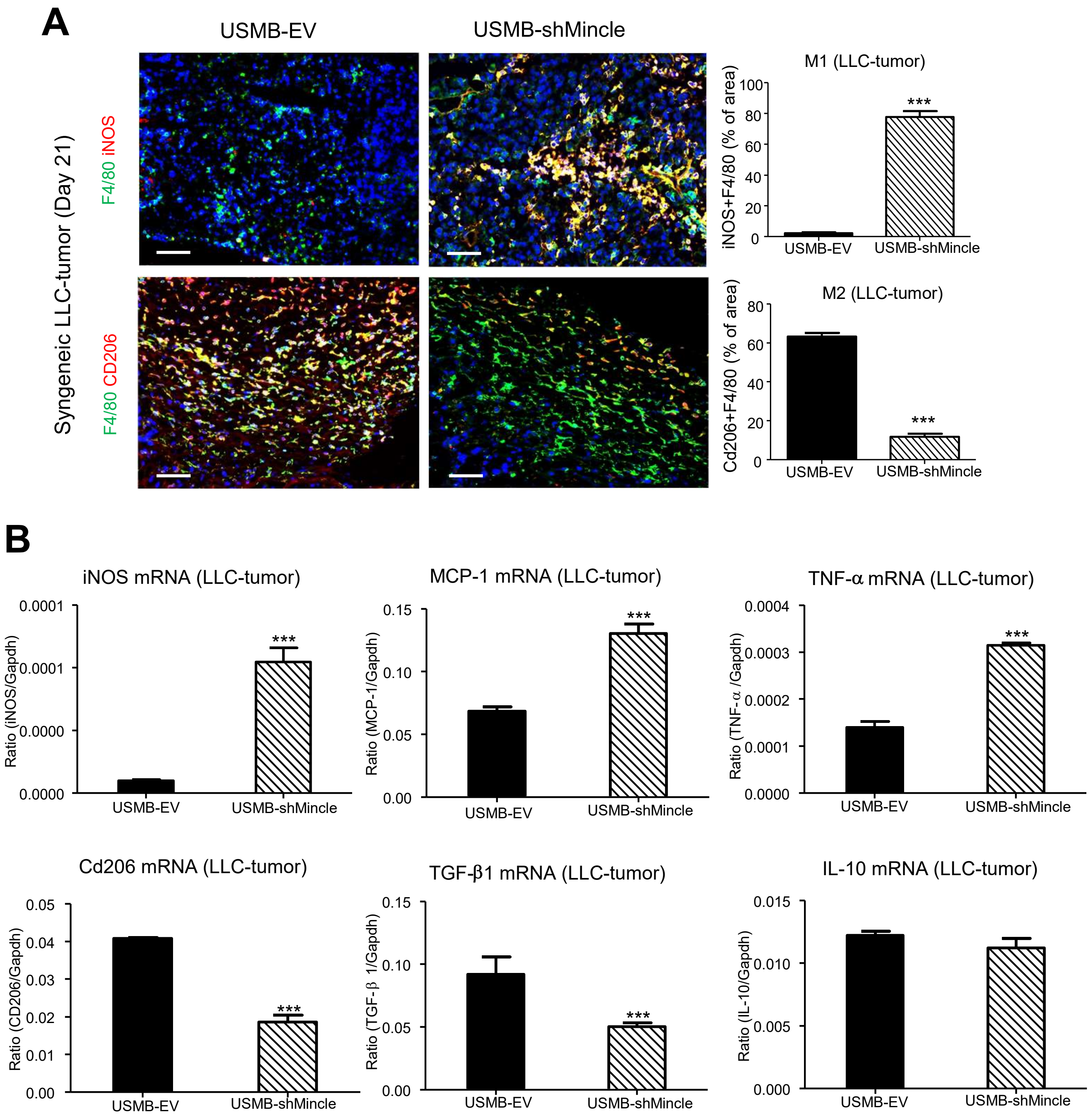

Figure S8. Effects of USMB-shMincle on TAM phenotypes and functions in syngeneic mouse lung carcinoma model LLC in vivo. (A) Immunofluorescence and **(B)** real-time PCR detects a significant increments of M1 (iNOS, MCP-1, TNF-α) but mild reduction or insignificant change of M2 (CD206, TGF-β1, IL-10) phenotypes and effectors in the LLC-tumor of immunocompetent C57BL/6J mice received USMB-shMincle compared to the mice received USMB-EV *in vivo*. Data represent mean ± SEM of 5 mice/group. *** $p < 0.001$ vs USMB-EV. Scale bars, 50 μm.

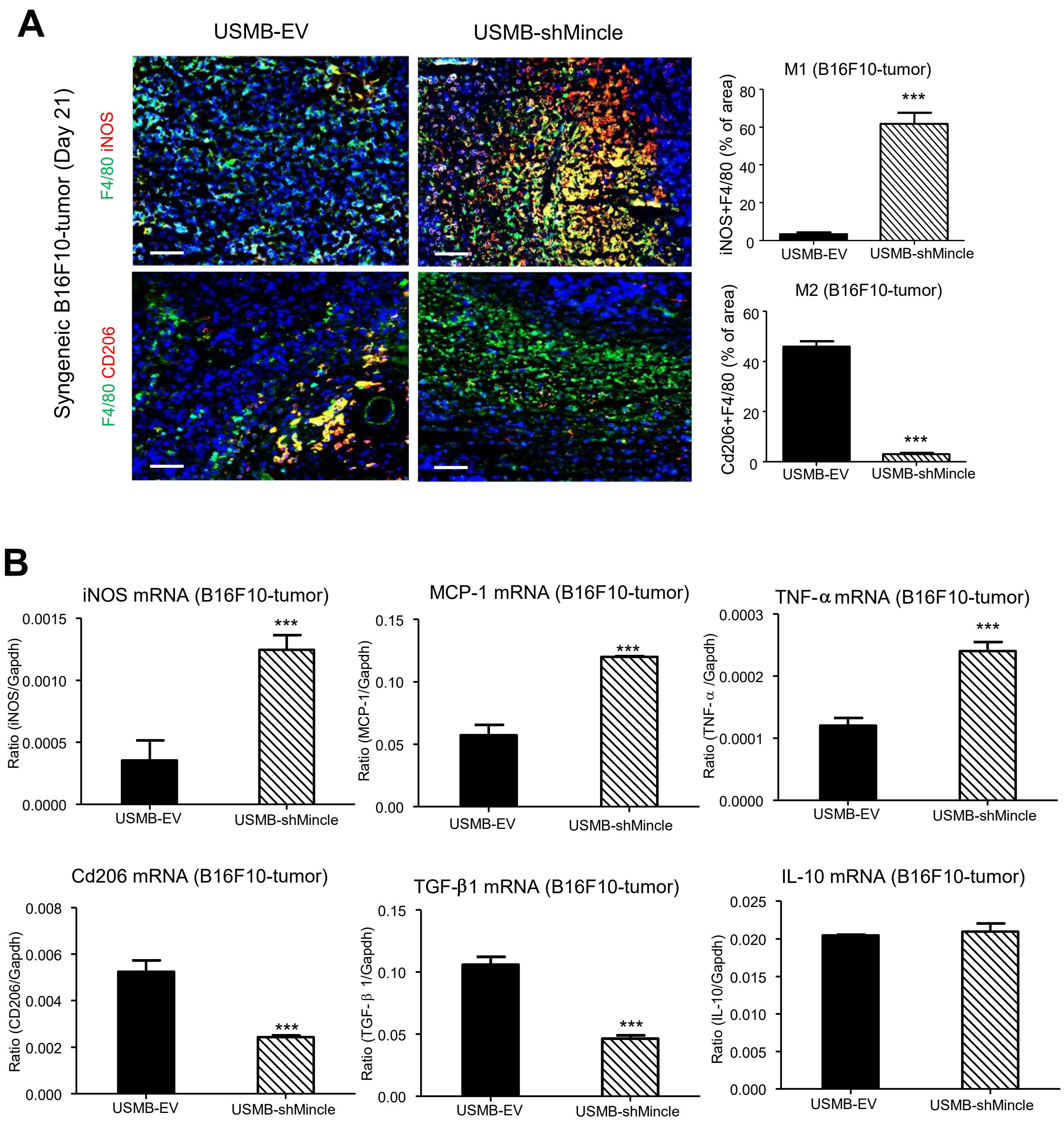

Figure S9. Effects of USMB-shMincle on TAM phenotypes and functions in syngeneic mouse melanoma model B16F10 in vivo. (A) Immunofluorescence and **(B)** real-time PCR detects a significant increments of M1 (iNOS, MCP-1, TNF-α) but mild reduction or insignificant change of M2 (CD206, TGF-β1, IL-10) phenotypes and effectors in the B16F10-tumor of immunocompetent C57BL/6J mice received USMB-shMincle compared to the mice received USMB-EV *in vivo*. Data represent mean ± SEM of 5 mice/group. *** $p < 0.001$ vs USMB-EV. Scale bars, 50 μm.

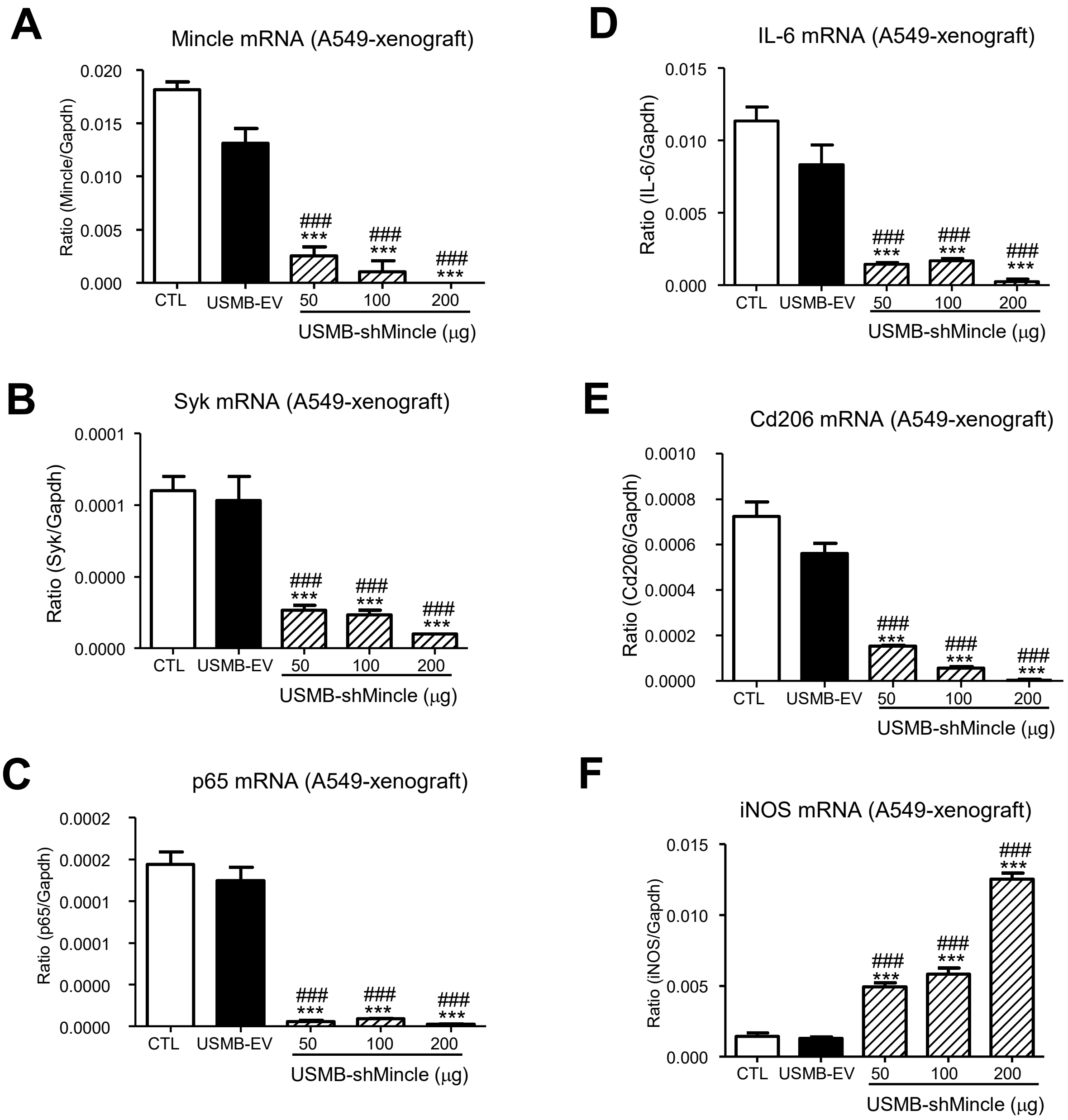

Figure S10. USMB-shMincle effectively blocks Mincle/Syk/NF-kB circuit and M1/M2 transition in A549-xenografts in vivo. Real-time PCR analysis showed that USMB-shMincle dose-dependently inhibits **(A-C)** Mincle/Syk/NF-kB circuit, **(D)** its protumoral effector IL-6, and **(E)** M2 phenotype Cd206 in the treated human NSCLC A549-xenografts. **(F)** Additionally, USMB-shMincle largely enhanced the M1 phenotype in the treated mice *in vivo*. Data represent mean ± SEM of 5 mice/group. Statistical analysis based on ordinary one-way ANOVA, *** $p < 0.001$ vs. CTL, ### $p < 0.001$ vs. EV.
