# Supplementary material for: USMB-shMincle: a virus-free gene therapy for blocking M1/M2 polarization of tumor-associated macrophages
Source: Mol Ther Oncolytics. 2021 Aug 25;23:26–37. doi: 10.1016/j.omto.2021.08.010 (PMC8463747; doi:10.1016/j.omto.2021.08.010)
Supplement: Document 2. Article plus supplemental information [file mmc2.pdf]

Molecular Therapy
Oncolytics
Original Article

USMB-shMincle: a virus-free gene therapy for blocking M1/M2 polarization of tumor-associated macrophages

Vivian Weiwen Xue,[1,8] Jeff Yat-Fai Chung,[1,8] Philip Chiu-Tsun Tang,[1] Alex Siu-Wing Chan,[2] Travis Hoi-Wai To,[1] Justin Shing-Yin Chung,[1] Francis Mussal,[3] Eric W.-F. Lam,[4] Chunjie Li,[5] Ka-Fai To,[1] Kam-Tong Leung,[6] Hui-Yao Lan,[7] and Patrick Ming-Kuen Tang[1]

[1]Department of Anatomical and Cellular Pathology, State Key Laboratory of Translational Oncology, The Chinese University of Hong Kong, Shatin 999077, Hong Kong; [2]Department of Applied Social Sciences, The Hong Kong Polytechnic University, Shatin 999077, Hong Kong; [3]Paediatric Oncology, Birmingham Children's Hospital, University of Birmingham, Birmingham B15 2TT, UK; [4]Sun Yat-sen University Cancer Center, State Key Laboratory of Oncology in South China, Collaborative Innovation Center for Cancer Medicine, Guangdong 510060, China; [5]Department of Head and Neck Oncology, West China Hospital of Stomatology, Sichuan University, Chengdu, Sichuan 610041, China; [6]Department of Paediatrics, The Chinese University of Hong Kong, Shatin 999077, Hong Kong; [7]Department of Medicine and Therapeutics, Li Ka Shing Institute of Health Sciences, The Chinese University of Hong Kong, Shatin 999077, Hong Kong

Mincle is essential for tumor-associated macrophage (TAM)-driven cancer progression and represents a potential immunotherapeutic target for cancer. Nevertheless, the lack of a specific inhibitor has largely limited its clinical translation. Here, we successfully developed a gene therapeutic strategy for silencing Mincle in a virus-free and tumor-specific manner by combining RNA interference technology with an ultrasound-microbubble-mediated gene transfer system (USMB). We identified a small hairpin RNA (shRNA) sequence shMincle that can silence not only Mincle expression but also the protumoral effector production in mouse bone marrow- and human THP-1-derived macrophages in the cancer setting *in vitro*. By using our well-established USMB system (USMB-shMincle), the shMincle-expressing plasmids were delivered in a tissue-specific manner into xenografts of human lung carcinoma A549 and melanoma A375 *in vivo*. Encouragingly, we found that USMB-shMincle effectively inhibited the protumoral phenotypes of TAMs as well as the progression of both A549 and A375 xenografts in a dose-dependent manner in mice without significant side effects. Mechanistically, we identified that USMB-shMincle markedly enhanced the anticancer M1 phenotype of TAMs in the A549 and A375 xenografts by blocking the protumoral Mincle/Syk/nuclear factor κB (NF-κB) signaling axis. Thus, USMB-shMincle may represent a clinically translatable novel and safe gene therapeutic approach for cancer treatment.

INTRODUCTION

Cancer is one of the top leading causes of death worldwide. However, ineffective treatments due to severe side effects, drug resistance, recurrence, and metastasis are the main difficulties encountered during clinical cancer treatment. Increasing evidence has shown that tumor microenvironment (TME) plays a vital role in tumor development and progression. Therefore, targeting TME is a promising approach for cancer treatment. In the past decade, a number of cell-based immunotherapeutic approaches, including chimeric antigen receptor (CAR) T cells and natural killer (NK) cells, have shown considerable progress in clinical trials.[1,2] Meanwhile, our recent studies also demonstrated that TME is essential for tumor growth, invasion, and metastasis via increasing angiogenesis and matrix metalloprotease (MMP) production and restricting NK cell cancer-killing activities.[3] These studies suggest that targeting TME may represent a safe and effective therapeutic strategy for cancer.

Tumor-associated macrophages (TAMs) are a type of predominant tumor-infiltrating leukocytes. Their role has been identified to be promotion of cancer progression via immunomodulation. Thus, the blockade of TAM activation and function is an important therapeutic strategy for TME-based anticancer therapy. The function of C-Type Lectin Domain Family 4 Member E (Clec4e, also called Mincle) in macrophages has attracted mounting attention recently. For example, Mincle was found to play an important role in inflammation-related diseases, such as acute renal inflammation and subarachnoid hemorrhage.[4,5] Unexpectedly, its potential role in TAMs is still largely unexplored. It has been demonstrated that deletion of Mincle markedly suppressed pancreatic tumorigenesis in a mouse model by enhancing

Received 20 May 2020; accepted 17 August 2021;
https://doi.org/10.1016/j.omto.2021.08.010.

[8]These authors contributed equally

Correspondence: Dr. Patrick Ming-Kuen Tang, Department of Anatomical and Cellular Pathology, State Key Laboratory of Translational Oncology, The Chinese University of Hong Kong, Shatin 999077, Hong Kong.
E-mail: patrick.tang@cuhk.edu.hk
Correspondence: Professor Hui-Yao Lan, Department of Medicine and Therapeutics, Li Ka Shing Institute of Health Sciences, The Chinese University of Hong Kong, Shatin 999077, Hong Kong.
E-mail: hylan@cuhk.edu.hk

 © 2021 The Author(s).
This is an open access article under the CC BY license (http://creativecommons.org/licenses/by/4.0/).

immunogenicity of the inflammatory TME including TAMs.[6] In addition, our previous study discovered that Mincle is essential for maintaining the protumoral activities of TAMs by suppressing the M1 phenotypes in two syngeneic mouse models with murine melanoma B16F10 and lung carcinoma LLC.[7] These findings suggested that Mincle is a pivotal gene for TAM-driven disease progression and a novel immunotherapeutic target for human cancer. Nevertheless, there is still no specific Mincle inhibitor available, which precludes its clinical transition.

Here, we successfully developed a novel virus-free gene therapy, USMB-shMincle, for tumor-specifically targeting Mincle by combining RNA interference (RNAi) technology with an ultrasound-microbubble (USMB)-mediated delivery system. Different from viral vectors, USMB-mediated delivery results in less safety and controllability concern in the clinical application of cancer treatment. Compared with non-viral systems, viral vectors have higher immunogenicity and genotoxicity, which can affect treatment efficiency.[8] For example, lentivirus-mediated gene therapy integrates therapeutic DNA sequences into the host genome, while USMB-mediated RNAi as a non-viral gene therapy is more controllable in treatment time and dosage.[9]

In this study, we further optimize the USMB-shMincle system for targeting both mouse and human Mincle-expressing TAMs *in vitro* and *in vivo*. The translational potential and safety of the modified system were evaluated by using two human xenograft cancer models with invasive melanoma A375 and lung cancer A549. Encouragingly, USMB-shMincle treatment not only dose-dependently blocked the M1/M2 polarization but also reduced the production of TAM-mediated protumoral effectors in the human xenografts *in vivo*. This pre-clinical study provides important rationales for further developing USMB-shMincle as a novel and safe therapeutic strategy for human cancers.

RESULTS

Screening for a universal shRNA that silences both human and mouse Mincle

To develop a clinical translatable therapeutic strategy, we first designed three small interfering RNAs (siRNAs) according to the conserved sequences of murine and human Mincle mRNA for achieving universal Mince knockdown (Table S1). The silencing efficiencies of these siRNAs were tested on mouse macrophage cell line RAW264.7 and THP-1-derived human macrophages. In essence, both siMincle #2 and #3 effectively silenced Mincle expression in cancer secretome-primed macrophage cell lines RAW264.7 and THP-1, under stimulation with cancer conditional medium (CM), as revealed by real-time PCR and western blot analyses (Figures 1A–1D). Interestingly, siMincle #3 in general achieved the largest suppressive effects on Mincle transcription in mouse primary cultured bone marrow-derived macrophages (BMDMs) and both RAW264.7 and THP-1 macrophage cell lines under lipopolysaccharide (LPS) stimulation compared with the other two siMincle sequences (Figure S1). As siMincle #3 effectively inhibited both human and mouse Mincle expression at both the mRNA and protein levels, it was converted to a small hairpin RNA (shRNA) sequence and cloned into an expression plasmid as shMincle for further investigation (Figures 1E and 1F).

shMincle effectively inhibits protumoral activities of macrophage *in vitro*

We confirmed further that shMincle was able to suppress the expression of Mincle under stimulations of LPS or CM, as the siMincle #3 siRNA *in vitro* (Figures 2A and S2). More importantly, we observed that shMincle effectively inhibited not only Mincle expression but also interleukin 6 (IL-6) production in the CM-induced THP-1 and RAW264.7 macrophages in a dose-dependent manner *in vitro* (Figures 2B–2G). Notably, IL-6 is a key protumoral cytokine, whose production is regulated by the Mincle/Syk/nuclear factor κB (NF-κB) signaling circuit in TAMs,[7] suggesting that shMincle effectively inhibits this protumoral axis and may produce anti-cancer effects *in vivo*.

USMB-shMincle effectively inhibits cancer progression in human xenografts

To assess its therapeutic effects *in vivo*, shMincle was tumor-specifically delivered into two xenograft mouse models bearing human lung carcinoma (A549) and melanoma (A375) with our well-established USMB system.[10–12] In brief, when tumor sizes reached 50 mm^3 after cancer cell inoculation, the human xenograft-bearing mice were randomized and treated with empty vector (EV) or various dosages of shMincle (50 μg, 100 μg, and 200 μg/mouse) mixed with microbubble delivery reagent at 1:1 ratio every 5 days via tail vein injection. Encouragingly, USMB-shMincle effectively inhibited the progression of both human lung carcinoma and melanoma *in vivo*. In more detail, the growth of human lung carcinoma A549 xenografts was dose-dependently suppressed by USMB-shMincle compared to the control group, whereas no significant effects were found on the mice treated with EV (USMB-EV, Figures 3A–3C). Interestingly, the expression of Mincle in TAMs was largely repressed at the dosages of 100 μg/mouse and above in the TME (Figure 3D). In addition, USMB-shMincle also significantly inhibited the growth of human melanoma A375 xenografts in mice, with a marked reduction of Mincle-expressing TAMs (Figures 4A–4D). It is noteworthy that the groups receiving high dosages of USMB-shMincle (200 μg/mouse) showed 60% and 75% regression of A375 and A549 xenografts compared to their USMB-EV controls, suggesting that USMB-shMincle may represent an effective gene therapeutic approach for human cancer.

USMB-shMincle is a safe anticancer gene therapy

Importantly, we evaluated the safety of USMB-shMincle therapy by evaluating its potential cytotoxic effects on the important organs of the treated A375- and A549-bearing mice. By conducting histological evaluation with hematoxylin and eosin (H&E) stain, we observed no significant damage in the heart, liver, kidney, and spleen of the USMB-shMincle-treated mice compared to the control groups (Figures 5A and S3). More specifically, we also examined the potential side effects of USMB-shMincle by enzymatic method through enzyme-linked immunosorbent assay (ELISA). Our data suggested that there were no significant increments of the serum levels of tissue-specific enzymes including alanine aminotransferase (ALT),

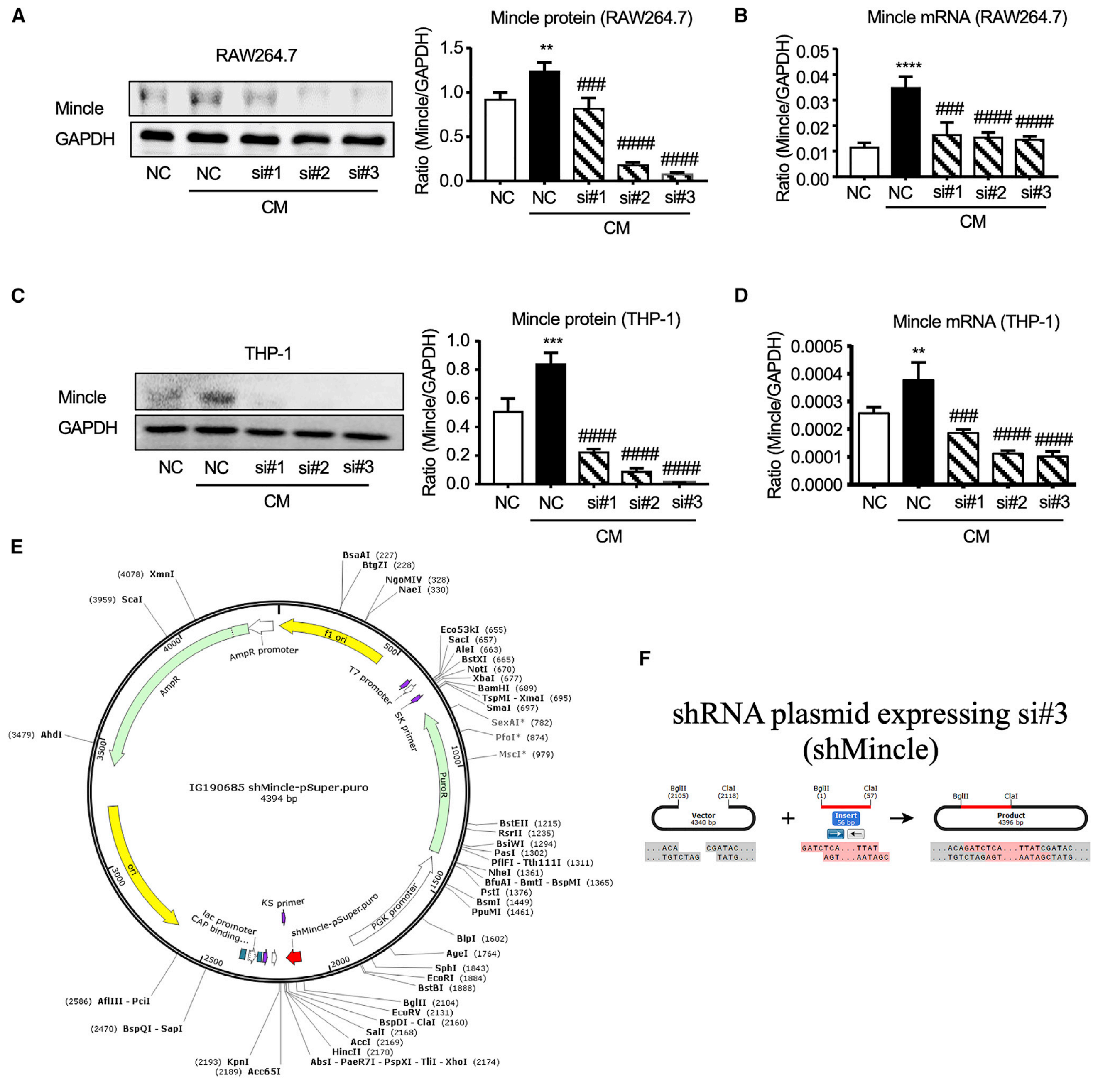

Figure 1. Screening for universal shMincle sequences in human and mouse macrophages

Knockdown efficiencies of three designed sequences against Mincle in (A and B) murine macrophage RAW264.7 and (C and D) human macrophage phorbol 12-myristate 13-acetate (PMA)-stimulated THP-1 under cancer secretome stimulation *in vitro*. siMincle #3 showed the best silencing for Mincle in both human and murine macrophages. (E and F) Map of constructed shMincle plasmid using si#3 and pSUPER.puro vector. Data represent mean ± SEM of 3 independent *in vitro* experiments. Statistical analysis based on ordinary one-way ANOVA. ****p < 0.0001, ***p < 0.001, **p < 0.01 versus NC; ####p < 0.0001, ###p < 0.001 versus NC+CM.

aspartate aminotransferase (AST), lactate dehydrogenase (LDH), myeloperoxidase (MPO), creatinine, and troponin-1 (Tn-1) in the USMB-shMincle-treated mice compared to the untreated and EV controls (Figures 5B–5G and S3), highlighting that USMB-shMincle is a safe gene therapy for cancer treatment.

USMB-shMincle enhances M1 TAMs by blocking the Mincle/Syk/NF-κB axis

We recently identified that the novel Mincle/Syk/NF-κB circuit is important for promoting the protumoral activities of TAMs by facilitating their M1/M2 transition, which can be blocked

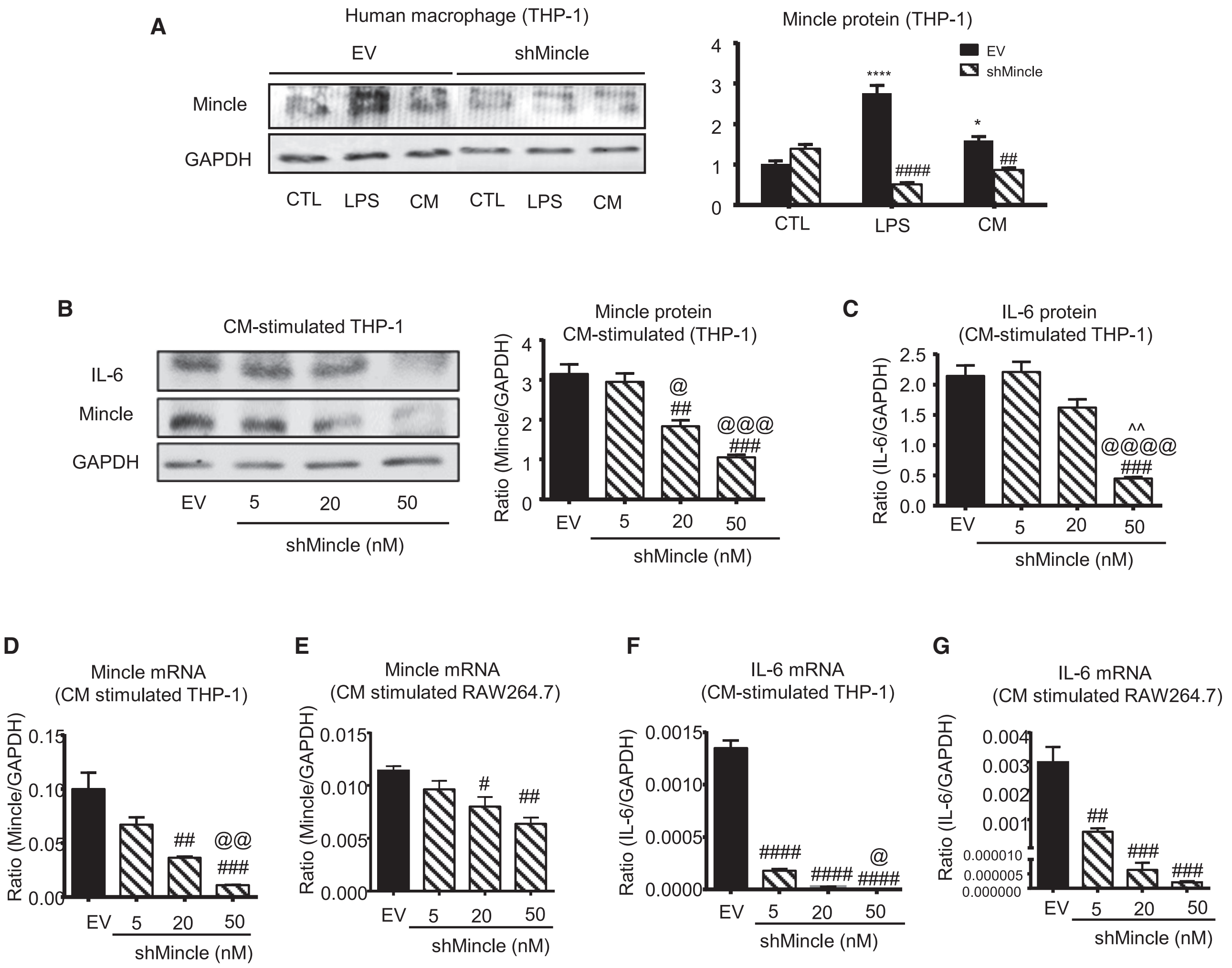

Figure 2. shMincle effectively inhibits Mincle expression in human macrophages

(A) shMincle effectively inhibited Mincle expression in human macrophage THP-1 cells upon both LPS and CM stimulation. (B–G) shMincle blocks Mincle and downstream IL-6 expression in a dose-dependent manner in human THP-1 and murine RAW264.7 macrophages. Data represent mean ± SEM of 3 independent *in vitro* experiments. Statistical analysis based on ordinary one-way or two-way ANOVA. ####p < 0.0001, ###p < 0.001, ##p < 0.01, #p < 0.05 versus EV; @@@@p < 0.0001, @@@p < 0.001, @@p < 0.01, @p < 0.05 versus 5 nM shMincle; ^^p < 0.01 versus 20 nM shMincle.

by targeting Mincle.[7] Consistently, we found that USMB-shMincle effectively suppressed the M1/M2 transition in A549 and A375 xenografts, as shown by the dose-dependently enhanced M1 but suppressed M2 phenotypes and functions without change in total TAM number in the treated xenografts (Figures 6A–6C, S4, S5, S6, and S7). It should be noted that the same inhibitory effect on M1/M2 polarization was also detected in the immunocompetent mouse models with syngeneic lung carcinoma LLC and melanoma B16F10 *in vivo* (Figures S8 and S9). Additionally, USMB-shMincle not only substantially blocked the Mincle/Syk/NF-κB circuit and its key protumoral cytokine IL-6 (Figures 6E and 6F, S5, and S10) but also greatly reduced the production of invasive and metastatic effectors CXCR4 and MMP-13 (Figure 7) in the shMincle xenografts at both mRNA and protein levels *in vivo*. Our results demonstrated that USMB-shMincle significantly inhibited the malignant progression of human melanoma and lung carcinoma in mouse xenograft models by blocking M1/M2 transition and protumoral effector production in a tumor-specific manner. Thus, USMB-shMincle may represent as an effective and safe virus-free gene therapy for human cancers.

DISCUSSION

In our previous studies, we discovered that Mincle is highly expressed in TAMs and has an essential role in promoting the protumoral activities of TME via a novel Mincle/Syk/NF-κB signaling circuit.[7] Nevertheless, there is still no specific Mincle inhibitor available worldwide, which greatly restricts its potential for clinical translation. Here, we

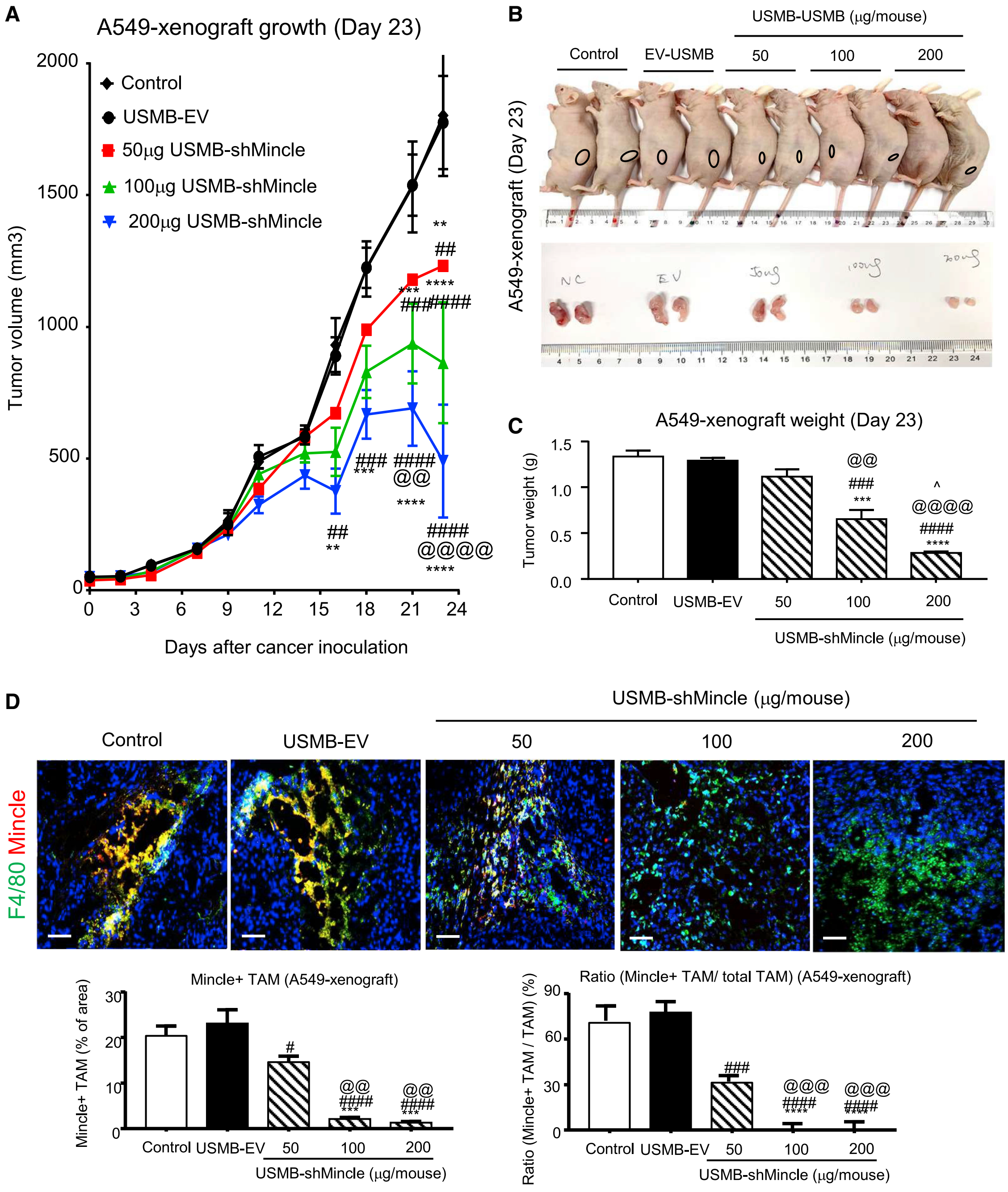

Figure 3. USMB-shMincle effectively blocks the growth of human non-small-cell lung carcinoma (NSCLC) xenografts *in vivo*

Mice were inoculated with 1 × 10^6 human lung cancer A549 cells subcutaneously and then received USMB-mediated tumor-specific delivery with 100 µL of mixture with EV (USMB-EV) or shMincle plasmid (50 µg, 100 µg, and 200 µg/mouse; USMB-shMincle) every 5 days as treatment. (A–C) Imaging of A549 human lung cancer-bearing mice and tumors, tumor weight, and tumor size. (D) Mincle expression in TAMs was highly suppressed in 100-µg and 200-µg dosages of USMB-shMincle treatment. Data represent mean ± SEM of 5 mice/group. Statistical analysis based on ordinary one-way ANOVA. ****p < 0.0001, ***p < 0.001 versus Control; ####p < 0.0001, ###p < 0.001, #p < 0.05 versus EV-USMB; @@@@p < 0.0001, @@@p < 0.001, @@p < 0.01 versus 50 µg shMincle; ^p < 0.05 versus 100 µg shMincle. Scale bars, 100 µm.

established a virus-free gene therapy, USMB-shMincle, as a novel Mincle inhibitor and successfully demonstrated its anticancer efficiency and safety in two xenograft nude mouse models of human melanoma and lung carcinoma. This work evidently demonstrated that USMB-shMincle may represent a potential anticancer gene therapy for human cancers.

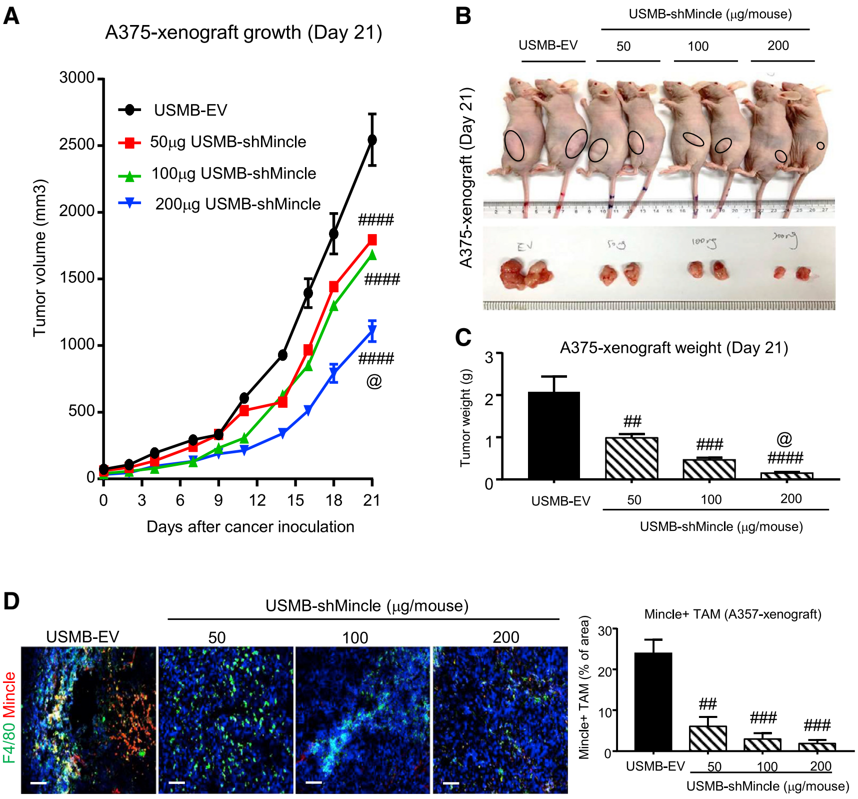

Figure 4. USMB-shMincle effectively blocks the growth of human melanoma xenografts *in vivo*

Mice were inoculated with 1 × 10^6 human melanoma A375 cells subcutaneously and then received 100 µL of USMB mixture with EV or shMincle plasmid (50 µg, 100 µg, and 200 µg/mouse) every 5 days as treatment. (A–C) Imaging of A375 human melanoma-bearing mice and tumors, tumor weight, and tumor size. (D) Mincle expression in TAMs was highly suppressed under USMB-shMincle treatment. Data represent mean ± SEM of 5 mice/group. Statistical analysis based on ordinary one-way ANOVA. ####$p < 0.0001$, ###$p < 0.001$, ##$p < 0.01$ versus EV; @$p < 0.05$ versus 50 µg shMincle. Scale bars, 100 µm.

The role of Mincle was initially reported in primary immunity to microorganisms and inflammation. In 2009, Yamasaki et al. reported that Mincle senses damaged cells and Mincle$^{-/-}$ macrophages have defects in cytokine and chemokine production under pathogenic fungal infection.[13] The classic activation pathway of Mincle is to cross-link the tyrosine activation motif ITAM subunit FcRγ after binding to ligands such as LPS and trehalose-6,6′-dimycolate (TDM),[14] which recruits Syk through phosphorylation and causes the CARD9-MALT1-BCL10 complex to activate the NF-κB pathway and release cytokines.[15,16] These series of reactions invoke immune cell infiltration[6] and activation.[17] In addition, Mincle$^{-/-}$ bone marrow-derived cells lose their ability in dynamic balance of pro- and anti-inflammatory cytokines, which affects cytotoxicity $CD4^+$ T cell function such as interferon (IFN)-γ production.[18] Glycolipid-stimulated M1 polarization is another important immunomodulation in phagocytosis.[19] Moreover, increasing evidence has indicated that Mincle functions in diseases including acute kidney injury and cancers. In acute kidney injury, Mincle is essential for maintaining M1 phenotype of macrophages, which promotes cisplatin-induced renal inflammation.[4] On the other hand, Mincle is an important immunoregulator in cancer immunity. In 2016, Seifert et al. discovered that Mincle binds to the endogenous ligand SAP130 released during cell necrosis, which increases the infiltration of M2-type TAMs and myeloid-derived suppressor cells (MDSCs) and inhibits cytotoxic T cell function as well.[6] Gene therapy targeting Mincle in TAMs provides new ideas for cancer immunotherapy. Unexpectedly, we uncovered a therapeutic effect of USMB-shMincle in blocking the M1/M2 transition. Importantly, M2 TAMs promote angiogenesis,[20–23] immunosuppressive environment, as well as generation of CD31-positive vessels during cancer development.[24] Similarly, higher vascular endothelial growth factor (VEGF) expression was also observed to be correlated with increased M2 TAM infiltration in bladder cancer.[25] Our newly invented USMB-shMincle may represent a potential immunotherapeutic strategy for eliminating M2 production in cancer. Undoubtedly, TAMs have complex regulation mechanisms. Apart from the Mincle signaling pathway, uptake of extracellular oncogenic protein $KRAS^{G12D}$[26] and apoptotic tumor cell-derived microRNAs (miRNAs),[27] hedgehog signaling activation,[28] and a high level of bcl-2 expression[29] also can affect TAM polarization. However, the interaction between the Mincle signaling pathway and other regulatory mechanisms is still unclear and needs further exploration.

Gene therapy is one of the potential solutions for cancer; however, safety concerns for the use of viruses still greatly limit its clinical application.[30] For example, an early clinical trial showed insertional oncogenesis in 4 patients who received retrovirus-mediated gene therapy of X-linked SCID, which led to serious adverse effects.[31] Although the latest self-inactivating lentiviral vectors have largely reduced the risks of mutagenesis, neighboring genes are still activated in some studies when viruses with strong promoters and enhancer elements are used.[32] In consequence, we combined RNAi with our well-established USMB gene transfer system, which has been studied intensively in kidney disease models.[10–12] As shRNA regulates gene expression at the transcriptional level without affecting the genome and USMB delivers the shMincle in a tumor-specific manner, we detected no off-target effects on important organs of the cancer host. These encouraging findings suggest that our newly invented USMB-shMincle is a safe therapeutic strategy for cancer. For treatment durability, direct genetic knockout has a longer-term therapeutic effect. In a recent phase I clinical trial, the

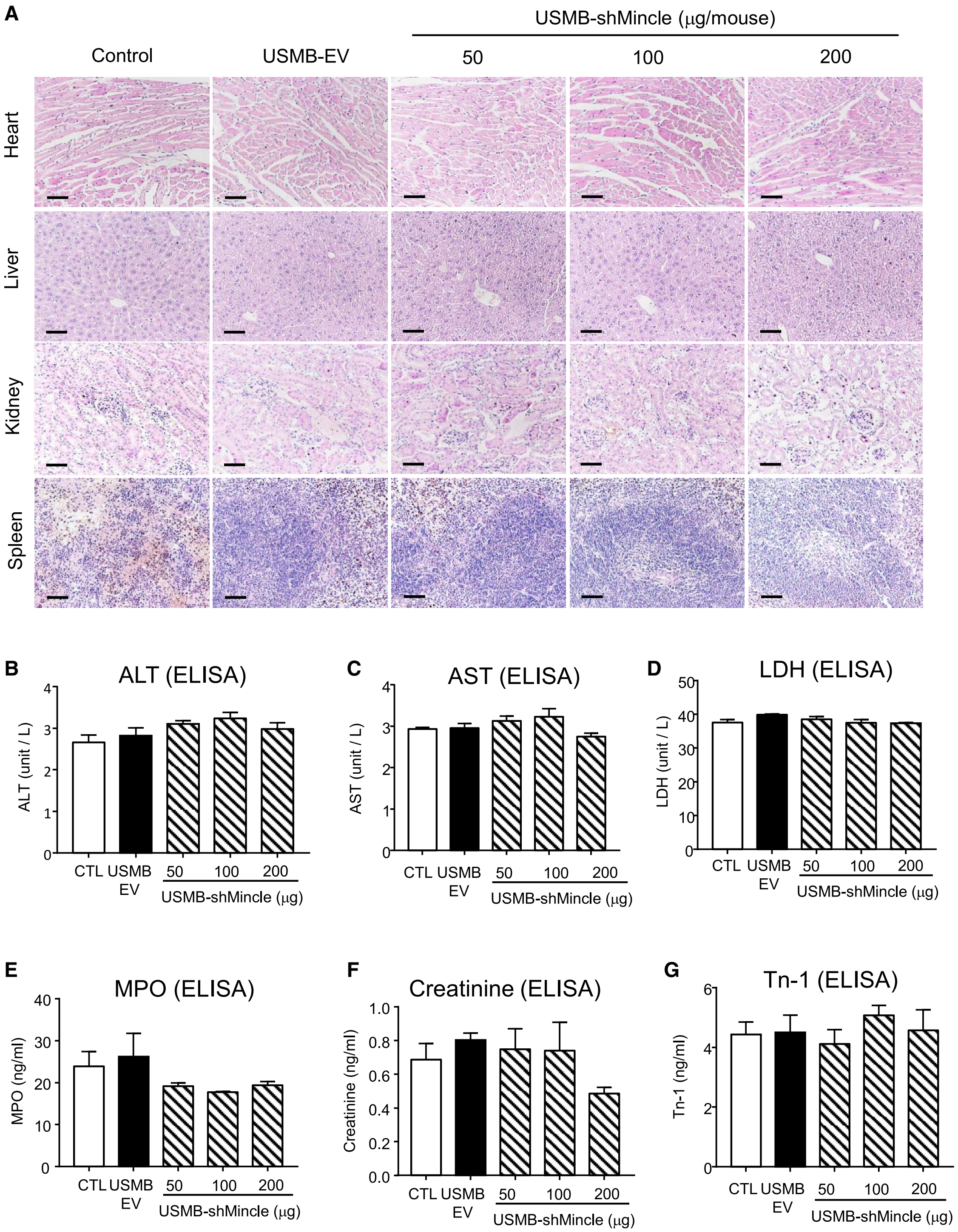

Figure 5. USMB-shMincle is a safe gene therapy without side effects

Serum samples and tissue sections from melanoma- and lung cancer-bearing mice were collected for safety evaluation of USMB-shMincle. (A) H&E staining of tissues from A549 human lung cancer-bearing mice. ALT (B), AST (C), LDH (D), MPO (E), creatinine (F), and Tn-1 (G) in serum samples were analyzed by ELISA. Data represent mean ± SEM of 5 mice/group. Scale bars, 100 μm.

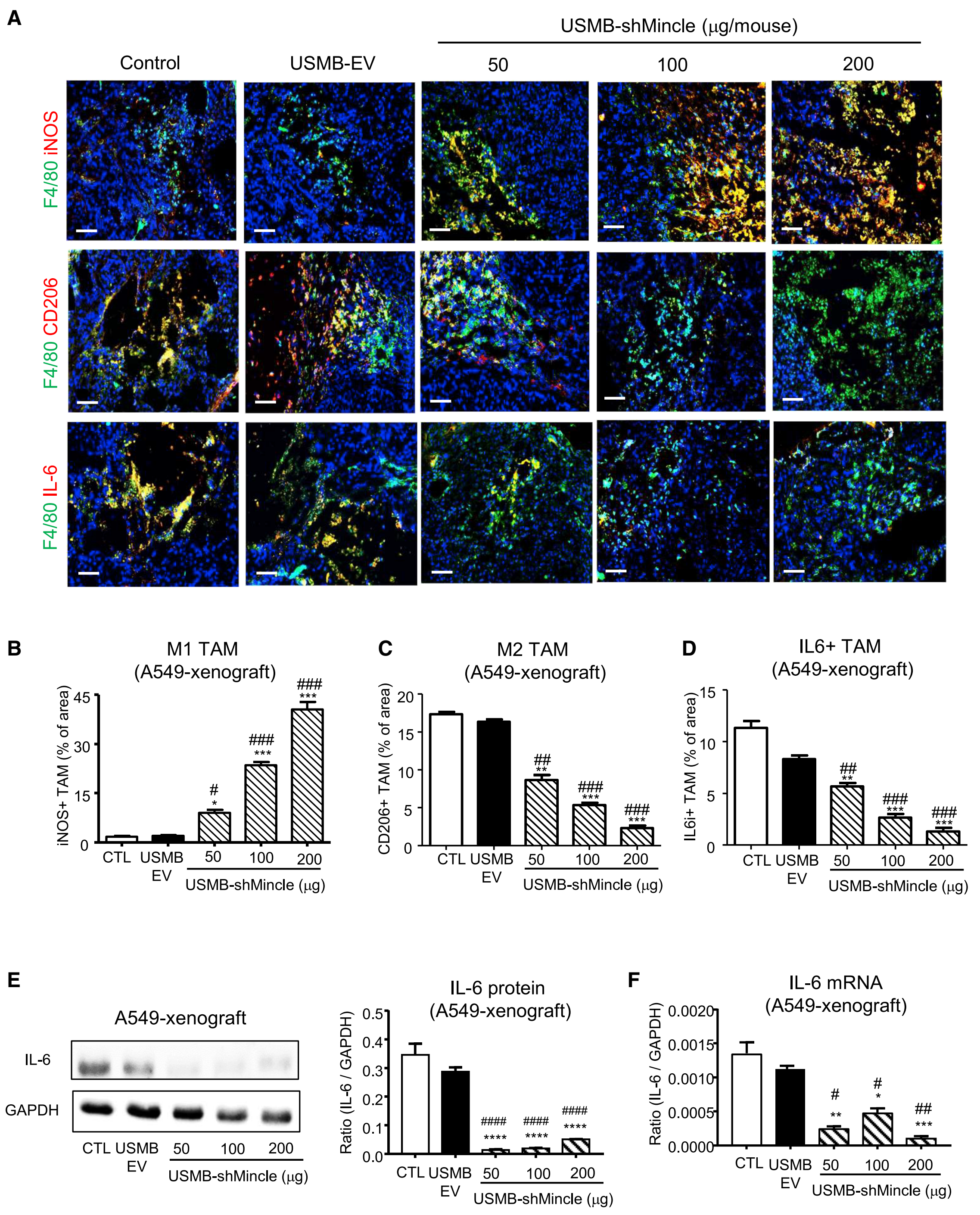

Figure 6. USMB-shMincle markedly inhibits M1/M2 transition of TAMs *in vivo*

(A–D) USMB-shMincle dramatically increased the M1 phenotype (iNOS) but decreased M2 phenotype (CD206) and the protumoral effector IL-6 in TAMs. (E and F) USMB-shMincle inhibited the production of Mincle/Syk/NF-κB downstream effector IL-6, detected in the A549 xenograft by western blot and real-time PCR. Data represent mean ± SEM of 5 mice/group. Statistical analysis based on ordinary one-way ANOVA. ****p < 0.0001, ***p < 0.001, **p < 0.01, *p < 0.05 versus CTL; ####p < 0.0001, ###p < 0.001, ##p < 0.01, #p < 0.05 versus EV. Scale bars, 100 μm.

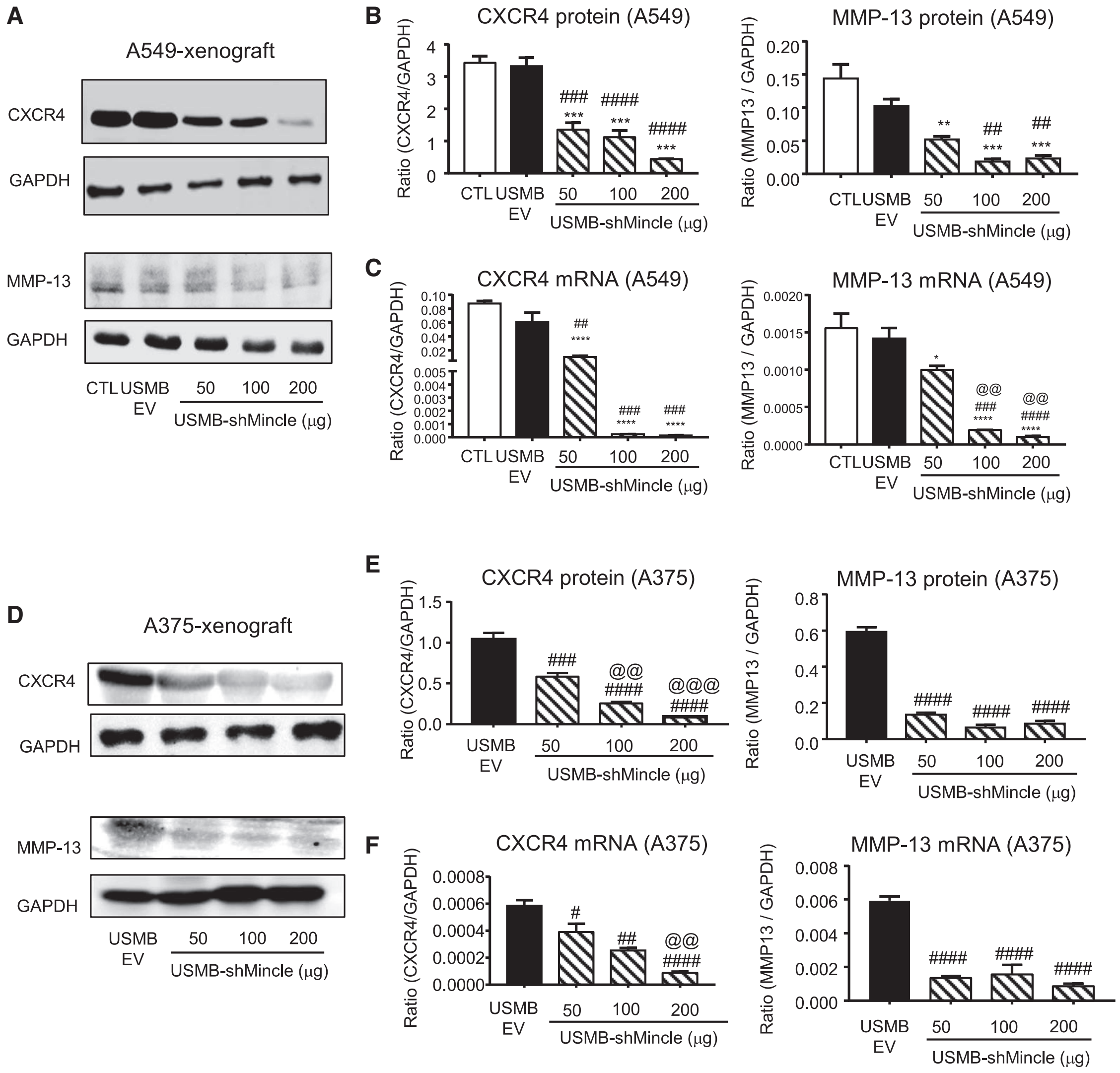

Figure 7. USMB-shMincle significantly suppresses production of invasion and metastasis efforts in TME *in vivo*

Interestingly, USMB-shMincle significantly inhibited the production of CXCR4 and MMP-13, important effectors for promoting cancer invasion and metastasis, in the human xenografts of lung carcinoma A549 (A–C) and melanoma A375 (D–F) *in vivo*. Data represent mean ± SEM of 5 mice/group. Statistical analysis based on ordinary one-way ANOVA. ****$p < 0.0001$, ***$p < 0.001$, **$p < 0.01$, *$p < 0.05$ versus CTL; ####$p < 0.0001$, ###$p < 0.001$, ##$p < 0.01$, #$p < 0.05$ versus USMB-EV; @@@$p < 0.001$, @@$p < 0.01$ versus 50 μg USMB-shMincle.

adoptive transfer of CRISPR-Cas9-engineered T cells that knocked out T cell receptor (TCR)α, TCRβ, and programmed cell death protein 1 (PD-1) could exist in patients for up to 9 months.[33] In comparison, inclisiran is a long-acting RNAi agent that provides inhibition on its target PCSK9 for more than 6 months.[34] We have detected that the USMB-mediated RNAi can significantly inhibit gene expression at day 7 after the first treatment, and the gene suppression could last at least 2 weeks *in vivo* in previous studies.[11,35] Similarly, shRNA-mediated gene inhibition will last several weeks *in vitro*.[36] Nevertheless, infiltration of TAMs into the TME is highly dynamic during cancer progression *in vivo*; we optimized the dosing to ensure the gene suppression effect according to our previous study.[9] Therefore, the optimized dosing schedule effectively achieved 70% knockdown efficiency *in vivo* similar to the lentiviral vectors.[37] Basically, USMB-shRNA is a non-viral cancer therapeutic strategy that provides competitive knockdown efficiency and higher safety and flexibility.

Additionally, we found that USMB-shMincle significantly inhibited CXCR4 and MMP-13 expression in both human melanoma and lung cancer mouse models. The high expression of MMP-13 has been reported to be commonly upregulated in most of the cancer microenvironments,[38] and increased stromal MMP-13 is important for promoting cancer invasion and angiogenesis.[39,40] In addition, CXCR4

has a role in cancer metastasis via activating the mitogen-activated protein kinase (MAPK) pathway.[41] Increased expression of CXCR4 in stromal cells and TME is also related to epithelial-mesenchymal transition and drug resistance.[42] The unexpected inhibitory effects of USMB-shMincle in the protumoral activities of TME should be investigated further, which may represent important information for its translational development.

The therapeutic principle of USMB-mediated gene transfer is to destroy the structure of the microbubble while effectively bringing the shRNA molecules carried by the microbubble into nearby cells.[43] Currently, some engineered microbubbles with pH/temperature-responsive and gas-generating features are expected to improve the targeting efficiency in cancer treatment.[44,45] However, USMB-mediated gene transfer does rely on ultrasound when targeting tumor sites, which focuses on localized drug delivery. This is a potential limitation for its use in the treatment of metastatic cancer, while USMB-mediated co-delivery of shRNA and chemotherapeutic drugs may provide a feasible way to apply the USMB-mediated treatment in aggressive and refractory cancers.[46] This highly targeted therapy also has many advantages, including that it does not affect the function of other organs. In immunotherapy, it targeted suppression of immune cells in TME without changing the baseline levels.

In summary, we successfully applied USMB-shMincle to develop a novel Mincle-targeted cancer immunotherapy in human invasive melanoma and lung cancer. Unexpectedly, we uncovered that the USMB-shMincle gene therapy also inhibits the M2 TAM-induced angiogenic and anti-apoptotic functions, which are important mechanisms that contribute to cancer progression mediated by TAMs. Thus, this pre-clinical study laid the foundation for Mincle-targeted cancer immunotherapy via USMB-shMincle.

MATERIALS AND METHODS

Animals

Nude mice and C57BL/6 mice (male, 8–10 week old) were purchased from the Chinese University of Hong Kong Laboratory Animal Services Centre. All experimental procedures were approved by the Animal Ethics Experimental Committee of the Chinese University of Hong Kong.

Cancer cell culture

Human A375 and A549 and mouse LLC and B16F10 cancer cells were cultured in DMEM and DMEM/F12 medium (Life Technologies), respectively, with 10% heat-inactivated fetal bovine serum (FBS) and 1% penicillin and streptomycin in 5% CO_2 at 37°C as in previous studies.[37] Cell lines were free of mycoplasma by culture with the antimicrobial reagent Normocin (InvivoGen) 2 weeks prior to experiments. To collect CM, the cell culture medium was removed at 80% cell confluence, and cancer cells were cultured with serum-free medium for 24 h after washing with PBS. CMs from cancer cell lines were collected and filtered with a 0.2-μm membrane sterile filter.

Primary culture of BMDMs

For primary culture of BMDMs, bone marrow cells were harvested from 8- to 10-week old C57BL/6J mice as in previous studies.[4,47,48] In brief, bone marrow cells were harvested from femur and tibia via fine dissection. Red blood cells were lysed, and the remaining bone marrow cells were cultured in DMEM/F12 medium with 10% heat-inactivated FBS, 1% penicillin and streptomycin, and 50 ng/mL macrophage colony-stimulating factor (M-CSF) (Invitrogen) in 5% CO_2 at 37°C. BMDMs were harvested after 7 days of M-CSF-mediated macrophage differentiation. For *in vitro* knockdown assays, 50 nM of nonsense control (NC) or siMincle #1–3 was transfected into BMDMs with Lipofectamine RNAiMAX (Invitrogen) 24 h prior to experiments.[48] In addition, different dosages of shMincle plasmids (5 nM, 20 nM, and 50 nM) were also used to perform USMB-shMincle therapy as previously described.[7,10]

Western blot analysis

Protein from cultured cells and tumor tissues was extracted with radioimmunoprecipitation assay (RIPA) lysis buffer. Western blot analysis was performed as previously described.[3,12] In brief, after nonspecific binding was blocked with 5% bovine serum albumin (BSA), membranes were incubated overnight at 4°C with primary antibodies against glyceraldehyde-3-phosphate dehydrogenase (GAPDH) (sc-32233, Santa Cruz), Mincle (sc-390806, Santa Cruz), IL-6 (sc-32296, Santa Cruz), MMP13 (sc-30073, Santa Cruz), and CXCR4 (AB1848, Chemicon) and IRDye800-conjugated secondary antibodies (Rockland Immunochemicals). Signals were detected by the Odyssey imaging system (LI-COR Biosciences), and results were further quantified by ImageJ. The ratio of protein expression level was normalized against GAPDH expression.

Real-time PCR analysis

Messenger RNA expression was quantified by real-time PCR using SYBR Green Supermix (Life Technologies, Carlsbad, CA, USA) with primers as previously described[3,47,49] and shown in Table S2. The relative expression of detected genes was normalized against internal control GAPDH and calculated by the $2^{-\Delta\Delta Ct}$ method.

Ultrasound microbubble-mediated gene transfer

The xenograft mouse models were induced by subcutaneous inoculation with 1×10^6 A375 or A549 cells in the back of 8-week-old male or female C57BL/6J mice. For tumor-specific Mincle knockdown *in vivo*, eight mice for each group were treated with pSUPER.puro EV or shMincle recombinant plasmid via ultrasound microbubble-mediated gene transfer according to a modified protocol from our previous studies.[7,10] In brief, each mouse was intravenously injected with 100 μL of mixed microbubble (SonoVue, Bracco Suisse, Switzerland) solution containing 100 μg of EV or shMincle and immediately treated with an ultrasound transducer (Therasonic, Electro Medical Supplies, Wantage, UK) directly placed on the skin of the back against the tumor with an output of 1 MHz at 2 W/cm^2 for a total of 5 min. To maintain the transgene expression levels, the mice were transfected with EV or shMincle plasmid every 5 days and sacrificed

on day 21 and day 23 in the A375 human melanoma mouse model and the A549 human lung cancer mouse model, respectively.

Histology and immunofluorescence

Formalin-fixed paraffin-embedded (FFPE) tissue sections (5-μm thickness) of heart, liver, kidney, and spleen tissues from tumor-bearing nude mice were prepared and stained with H&E staining. Immunofluorescence was performed on fresh tissue sections (5-μm thickness) from mouse tumors and spleen tissues stained with antibodies against Mincle, IL-6, inducible nitric oxide synthase (iNOS) (sc-7271, Santa Cruz), and CD206 (sc-58986, Santa Cruz) conjugated with secondary antibody (Alexa 546 and Alexa 488) as previously described.[48,49]

Enzyme-linked immunosorbent assay

Serum samples from tumor-bearing mice were collected to detect cytotoxicity indicators with an ELISA kit as previously described.[37] ALT (TR71121, Thermo Scientific), AST (TR70121, Thermo Scientific), LDH (J2380, Promega), MPO (EMMPO, Life Technologies), creatinine (ab65340, Abcam), and Tn-1 (NBP3-00484, Novus) were measured according to the instructions of the manufacturers.

Statistical analysis

Statistical analysis was performed in GraphPad Prism 5 (GraphPad Software, La Jolla, CA, USA). All data are presented as mean ± SEM. Statistical significance was determined by $p < 0.05$ in one-way or two-way ANOVA.

SUPPLEMENTAL INFORMATION

Supplemental information can be found online at https://doi.org/10.1016/j.omto.2021.08.010.

ACKNOWLEDGMENTS

This study was supported by Innovation and Technology Fund of Hong Kong (ITS/068/18, PiH/009/19, PiH/010/19, PiH/394/19, InP/008/19, InP/009/19, InP/159/19); Research Grants Council of Hong Kong (14106518, 14111019, 14111720); and The Chinese University of Hong Kong's Faculty Innovation Award (4620528) and Direct Grant for Research (4054510). We also appreciate the technical support from Core Utilities of Cancer Genomics and Pathobiology of Department of Anatomical and Cellular Pathology, CUHK.

AUTHOR CONTRIBUTIONS

V.W.X. and J.Y.-F.C. conducted the experiments and designed research. V.W.X. drafted the manuscript. J.Y.-F.C. and P.C.-T.T. prepared figures. T.H.-W.T. and J.S.-Y.C. assisted with the animal models. C.L., K.-F.T., and K.-T.L. advised the study. F.M., E.W.-F.L., and A.S.-W.C. edited the manuscript. H.-Y.L. and P.M.-K.T. supervised the study. J.Y.-F.C., P.C.-T.T., and P.M.-K.T. edited and revised the manuscript.

DECLARATION OF INTERESTS

The authors declare no competing interests.

REFERENCES

1. Ma, Y., Zhang, S., Fang, H., Yu, K., and Jiang, S. (2020). A phase I study of CAR-T bridging HSCT in patients with acute CD19^{+} relapse/refractory B-cell leukemia. Oncol. Lett. *20*, 20.

2. Nguyen, R., Wu, H., Pounds, S., Inaba, H., Ribeiro, R.C., Cullins, D., Rooney, B., Bell, T., Lacayo, N.J., Heym, K., et al. (2019). A phase II clinical trial of adoptive transfer of haploidentical natural killer cells for consolidation therapy of pediatric acute myeloid leukemia. J. Immunother. Cancer *7*, 81.

3. Tang, P.M., Zhou, S., Meng, X.M., Wang, Q.M., Li, C.J., Lian, G.Y., Huang, X.R., Tang, Y.J., Guan, X.Y., Yan, B.P., et al. (2017). Smad3 promotes cancer progression by inhibiting E4BP4-mediated NK cell development. Nat. Commun. *8*, 14677.

4. Lv, L.L., Tang, P.M., Li, C.J., You, Y.K., Li, J., Huang, X.R., Ni, J., Feng, M., Liu, B.C., and Lan, H.Y. (2017). The pattern recognition receptor, Mincle, is essential for maintaining the M1 macrophage phenotype in acute renal inflammation. Kidney Int. *91*, 587–602.

5. Xie, Y., Guo, H., Wang, L., Xu, L., Zhang, X., Yu, L., Liu, Q., Li, Y., Zhao, N., Zhao, N., et al. (2017). Human albumin attenuates excessive innate immunity via inhibition of microglial Mincle/Syk signaling in subarachnoid hemorrhage. Brain Behav. Immun. *60*, 346–360.

6. Seifert, L., Werba, G., Tiwari, S., Giao Ly, N.N., Alothman, S., Alqunaibit, D., Avanzi, A., Barilla, R., Daley, D., Greco, S.H., et al. (2016). The necrosome promotes pancreatic oncogenesis via CXCL1 and Mincle-induced immune suppression. Nature *532*, 245–249.

7. Li, C., Xue, V.W., Wang, Q.M., Lian, G.Y., Huang, X.R., Lee, T.L., To, K.F., Tang, P.M., and Lan, H.Y. (2020). The Mincle/Syk/NF-κB Signaling Circuit Is Essential for Maintaining the Protumoral Activities of Tumor-Associated Macrophages. Cancer Immunol. Res. *8*, 1004–1017.

8. Monjezi, R., Miskey, C., Gogishvili, T., Schleef, M., Schmeer, M., Einsele, H., Ivics, Z., and Hudecek, M. (2017). Enhanced CAR T-cell engineering using non-viral Sleeping Beauty transposition from minicircle vectors. Leukemia *31*, 186–194.

9. Espinoza, D.A., Fan, X., Yang, D., Cordes, S.F., Truitt, L.L., Calvo, K.R., Yabe, I.M., Demirci, S., Hope, K.J., Hong, S.G., et al. (2019). Aberrant Clonal Hematopoiesis following Lentiviral Vector Transduction of HSPCs in a Rhesus Macaque. Mol. Ther. *27*, 1074–1086.

10. Zhang, Y.Y., Tang, P.M., Tang, P.C., Xiao, J., Huang, X.R., Yu, C., Ma, R.C.W., and Lan, H.Y. (2019). LRNA9884, a Novel Smad3-Dependent Long Noncoding RNA, Promotes Diabetic Kidney Injury in *db/db* Mice via Enhancing MCP-1-Dependent Renal Inflammation. Diabetes *68*, 1485–1498.

11. Feng, M., Tang, P.M., Huang, X.R., Sun, S.F., You, Y.K., Xiao, J., Lv, L.L., Xu, A.P., and Lan, H.Y. (2018). TGF-β Mediates Renal Fibrosis via the Smad3-Erbb4-IR Long Noncoding RNA Axis. Mol. Ther. *26*, 148–161.

12. Sun, S.F., Tang, P.M.K., Feng, M., Xiao, J., Huang, X.R., Li, P., Ma, R.C.W., and Lan, H.Y. (2018). Novel lncRNA Erbb4-IR Promotes Diabetic Kidney Injury in *db/db* Mice by Targeting miR-29b. Diabetes *67*, 731–744.

13. Yamasaki, S., Matsumoto, M., Takeuchi, O., Matsuzawa, T., Ishikawa, E., Sakuma, M., Tateno, H., Uno, J., Hirabayashi, J., Mikami, Y., et al. (2009). C-type lectin Mincle is an activating receptor for pathogenic fungus, Malassezia. Proc. Natl. Acad. Sci. USA *106*, 1897–1902.

14. Miyake, Y., Toyonaga, K., Mori, D., Kakuta, S., Hoshino, Y., Oyamada, A., Yamada, H., Ono, K., Suyama, M., Iwakura, Y., et al. (2013). C-type lectin MCL is an FcRγ-coupled receptor that mediates the adjuvanticity of mycobacterial cord factor. Immunity *38*, 1050–1062.

15. Shenderov, K., Barber, D.L., Mayer-Barber, K.D., Gurcha, S.S., Jankovic, D., Feng, C.G., Oland, S., Hieny, S., Caspar, P., Yamasaki, S., Lin, X., et al. (2013). Cord factor and peptidoglycan recapitulate the Th17-promoting adjuvant activity of mycobacteria through mincle/CARD9 signaling and the inflammasome. J. Immunol. *190*, 5722–5730.

16. Zhao, X.Q., Zhu, L.L., Chang, Q., Jiang, C., You, Y., Luo, T., Jia, X.M., and Lin, X. (2014). C-type lectin receptor dectin-3 mediates trehalose 6,6¿-dimycolate (TDM)-induced Mincle expression through CARD9/Bcl10/MALT1-dependent nuclear factor (NF)-κB activation. J. Biol. Chem. *289*, 30052–30062.

17. Dyevoich, A.M., Disher, N.S., Haro, M.A., and Haas, K.M. (2020). A TLR4-TRIF-dependent signaling pathway is required for protective natural tumor-reactive IgM production by B1 cells. Cancer Immunol. Immunother. *69*, 2113–2124.

18. Prado Acosta, M., Goyette-Desjardins, G., Scheffel, J., Dudeck, A., Ruland, J., and Lepenies, B. (2021). S-Layer From *Lactobacillus brevis* Modulates Antigen-Presenting Cell Functions via the Mincle-Syk-Card9 Axis. Front. Immunol. *12*, 602067.

19. Kodar, K., Harper, J.L., McConnell, M.J., Timmer, M.S.M., and Stocker, B.L. (2017). The Mincle ligand trehalose dibehenate differentially modulates M1-like and M2-like macrophage phenotype and function via Syk signaling. Immun. Inflamm. Dis. *5*, 503–514.

20. Chen, Y., Zhang, L., Liu, W.X., and Wang, K. (2018). VEGF and SEMA4D have synergistic effects on the promotion of angiogenesis in epithelial ovarian cancer. Cell. Mol. Biol. Lett. *23*, 2.

21. Cristina, C., Perez-Millan, M.I., Luque, G., Dulce, R.A., Sevlever, G., Berner, S.I., and Becu-Villalobos, D. (2010). VEGF and CD31 association in pituitary adenomas. Endocr. Pathol. *21*, 154–160.

22. Chang, H.H., Cheng, Y.C., Tsai, W.C., and Chen, Y. (2020). PSMB8 inhibition decreases tumor angiogenesis in glioblastoma through vascular endothelial growth factor A reduction. Cancer Sci. *111*, 4142–4153.

23. Yang, C., Shi, S., Su, Y., Tong, J.S., and Li, L. (2020). P2X7R promotes angiogenesis and tumour-associated macrophage recruitment by regulating the NF-κB signalling pathway in colorectal cancer cells. J. Cell. Mol. Med. *24*, 10830–10841.

24. Ma, Y., He, S., Gao, A., Zhang, Y., Zhu, Q., Wang, P., Yang, B., Yin, H., Li, Y., Song, J., et al. (2020). Methylation silencing of TGF-β receptor type II is involved in malignant transformation of esophageal squamous cell carcinoma. Clin. Epigenetics *12*, 25.

25. Wang, L.A., Yang, B., Tang, T., Yang, Y., Zhang, D., Xiao, H., Xu, J., Wang, L., Lin, L., and Jiang, J. (2020). Correlation of APE1 with VEGFA and $CD163^{+}$ macrophage infiltration in bladder cancer and their prognostic significance. Oncol. Lett. *20*, 2881–2887.

26. Dai, E., Han, L., Liu, J., Xie, Y., Kroemer, G., Klionsky, D.J., Zeh, H.J., Kang, R., Wang, J., and Tang, D. (2020). Autophagy-dependent ferroptosis drives tumor-associated macrophage polarization via release and uptake of oncogenic KRAS protein. Autophagy *16*, 2069–2083.

27. Frank, A.C., Ebersberger, S., Fink, A.F., Lampe, S., Weigert, A., Schmid, T., Ebersberger, I., Syed, S.N., and Brüne, B. (2019). Apoptotic tumor cell-derived microRNA-375 uses CD36 to alter the tumor-associated macrophage phenotype. Nat. Commun. *10*, 1135.

28. Petty, A.J., Li, A., Wang, X., Dai, R., Heyman, B., Hsu, D., Huang, X., and Yang, Y. (2019). Hedgehog signaling promotes tumor-associated macrophage polarization to suppress intratumoral CD8+ T cell recruitment. J. Clin. Invest. *129*, 5151–5162.

29. Di Martile, M., Farini, V., Consonni, F.M., Trisciuoglio, D., Desideri, M., Valentini, E., D'Aguanno, S., Tupone, M.G., Buglioni, S., Ercolani, C., et al. (2020). Melanoma-specific bcl-2 promotes a protumoral M2-like phenotype by tumor-associated macrophages. J. Immunother. Cancer *8*, e000489.

30. Bulcha, J.T., Wang, Y., Ma, H., Tai, P.W.L., and Gao, G. (2021). Viral vector platforms within the gene therapy landscape. Signal Transduct. Target. Ther. *6*, 53.

31. Hacein-Bey-Abina, S., Garrigue, A., Wang, G.P., Soulier, J., Lim, A., Morillon, E., Clappier, E., Caccavelli, L., Delabesse, E., Beldjord, K., et al. (2008). Insertional oncogenesis in 4 patients after retrovirus-mediated gene therapy of SCID-X1. J. Clin. Invest. *118*, 3132–3142.

32. Zufferey, R., Dull, T., Mandel, R.J., Bukovsky, A., Quiroz, D., Naldini, L., and Trono, D. (1998). Self-inactivating lentivirus vector for safe and efficient in vivo gene delivery. J. Virol. *72*, 9873–9880.

33. Stadtmauer, E.A., Fraietta, J.A., Davis, M.M., Cohen, A.D., Weber, K.L., Lancaster, E., Mangan, P.A., Kulikovskaya, I., Gupta, M., Chen, F., et al. (2020). CRISPR-engineered T cells in patients with refractory cancer. Science *367*, eaba7365.

34. Fitzgerald, K., White, S., Borodovsky, A., Bettencourt, B.R., Strahs, A., Clausen, V., Wijngaard, P., Horton, J.D., Taubel, J., Brooks, A., et al. (2017). A Highly Durable RNAi Therapeutic Inhibitor of PCSK9. N. Engl. J. Med. *376*, 41–51.

35. Yu, J.W., Duan, W.J., Huang, X.R., Meng, X.M., Yu, X.Q., and Lan, H.Y. (2014). MicroRNA-29b inhibits peritoneal fibrosis in a mouse model of peritoneal dialysis. Lab. Invest. *94*, 978–990.

36. Ledger, S., Howe, A., Turville, S., Aggarwal, A., Savkovic, B., Ong, A., Wolstein, O., Boyd, M., Millington, M., Gorry, P.R., et al. (2018). Analysis and dissociation of anti-HIV effects of shRNA to CCR5 and the fusion inhibitor C46. J. Gene Med. *20*, e3006.

37. Wang, Q.M., Tang, P.M., Lian, G.Y., Li, C., Li, J., Huang, X.R., To, K.F., and Lan, H.Y. (2018). Enhanced Cancer Immunotherapy with Smad3-Silenced NK-92 Cells. Cancer Immunol. Res. *6*, 965–977.

38. Gobin, E., Bagwell, K., Wagner, J., Mysona, D., Sandirasegarane, S., Smith, N., Bai, S., Sharma, A., Schleifer, R., and She, J.X. (2019). A pan-cancer perspective of matrix metalloproteases (MMP) gene expression profile and their diagnostic/prognostic potential. BMC Cancer *19*, 581.

39. Lederle, W., Hartenstein, B., Meides, A., Kunzelmann, H., Werb, Z., Angel, P., and Mueller, M.M. (2010). MMP13 as a stromal mediator in controlling persistent angiogenesis in skin carcinoma. Carcinogenesis *31*, 1175–1184.

40. Salaün, M., Peng, J., Hensley, H.H., Roder, N., Flieder, D.B., Houlle-Crépin, S., Abramovici-Roels, O., Sabourin, J.C., Thiberville, L., and Clapper, M.L. (2015). MMP-13 In-Vivo Molecular Imaging Reveals Early Expression in Lung Adenocarcinoma. PLoS ONE *10*, e0132960.

41. Urosevic, J., Blasco, M.T., Llorente, A., Bellmunt, A., Berenguer-Llergo, A., Guiu, M., Cañellas, A., Fernandez, E., Burkov, I., Clapés, M., et al. (2020). ERK1/2 signaling induces upregulation of ANGPT2 and CXCR4 to mediate liver metastasis in colon cancer. Cancer Res. *80*, 4668–4680.

42. Zhang, F., Cui, J.Y., Gao, H.F., Yu, H., Gao, F.F., Chen, J.L., and Chen, L. (2020). Cancer-associated fibroblasts induce epithelial-mesenchymal transition and cisplatin resistance in ovarian cancer via CXCL12/CXCR4 axis. Future Oncol. *16*, 2619–2633.

43. Paefgen, V., Doleschel, D., and Kiessling, F. (2015). Evolution of contrast agents for ultrasound imaging and ultrasound-mediated drug delivery. Front. Pharmacol. *6*, 197.

44. Li, Z., Fan, F., Ma, J., Yin, W., Zhu, D., Zhang, L., and Wang, Z. (2021). Oxygen- and bubble-generating polymersomes for tumor-targeted and enhanced photothermal-photodynamic combination therapy. Biomater. Sci. *9*, 5841–5853.

45. Zhang, K., Xu, H., Chen, H., Jia, X., Zheng, S., Cai, X., Wang, R., Mou, J., Zheng, Y., and Shi, J. (2015). CO2 bubbling-based 'Nanobomb' System for Targetedly Suppressing Panc-1 Pancreatic Tumor via Low Intensity Ultrasound-activated Inertial Cavitation. Theranostics *5*, 1291–1302.

46. Bae, Y.J., Yoon, Y.I., Yoon, T.J., and Lee, H.J. (2016). Ultrasound-Guided Delivery of siRNA and a Chemotherapeutic Drug by Using Microbubble Complexes: In Vitro and In Vivo Evaluations in a Prostate Cancer Model. Korean J. Radiol. *17*, 497–508.

47. Tang, P.M., Zhou, S., Li, C.J., Liao, J., Xiao, J., Wang, Q.M., Lian, G.Y., Li, J., Huang, X.R., To, K.F., et al. (2018). The proto-oncogene tyrosine protein kinase Src is essential for macrophage-myofibroblast transition during renal scarring. Kidney Int. *93*, 173–187.

48. Tang, P.M., Zhang, Y.Y., Xiao, J., Tang, P.C., Chung, J.Y., Li, J., Xue, V.W., Huang, X.R., Chong, C.C., Ng, C.F., et al. (2020). Neural transcription factor Pou4f1 promotes renal fibrosis via macrophage-myofibroblast transition. Proc. Natl. Acad. Sci. USA *117*, 20741–20752.

49. Tang, P.M., Zhang, Y.Y., Hung, J.S., Chung, J.Y., Huang, X.R., To, K.F., and Lan, H.Y. (2021). DPP4/CD32b/NF-κB Circuit: A Novel Druggable Target for Inhibiting CRP-Driven Diabetic Nephropathy. Mol. Ther. *29*, 365–375.

OMTO, Volume *23*

Supplemental information

USMB-shMincle: a virus-free gene therapy for blocking M1/M2 polarization of tumor-associated macrophages

Vivian Weiwen Xue, Jeff Yat-Fai Chung, Philip Chiu-Tsun Tang, Alex Siu-Wing Chan, Travis Hoi-Wai To, Justin Shing-Yin Chung, Francis Mussal, Eric W.-F. Lam, Chunjie Li, Ka-Fai To, Kam-Tong Leung, Hui-Yao Lan, and Patrick Ming-Kuen Tang

Supplementary Table S1. Target sequences of the siRNA candidates.

siRNA	Target sequence
siMincle #1	GTCAGTGGCAATGGGTGGA
siMincle #2	GTTTCATCACCAGATGTGT
siMincle #3	CAGGTTCAGTCAAGAATTG

Supplementary Table S2. List of primers used in this study.

Mouse gene	Forward primer (5' to 3')	Reverse primer (5' to 3')
Gapdh	GCATGGCCTTCCGTGTTC	GATGTCATCATACTTGGCAGGTTT
Syk	CTACTACAAGGCCCAGACC	TGATGCATTCGGGGGCGTAC
P65	CGAATCCAGACCAACAATAACC	ACAGCAAGAAGATCTCATCCC
Cxcr4	TCCTCCTGACTATACCTGACTTCATCT	CCTGTCATCCCCCTGACTGAT

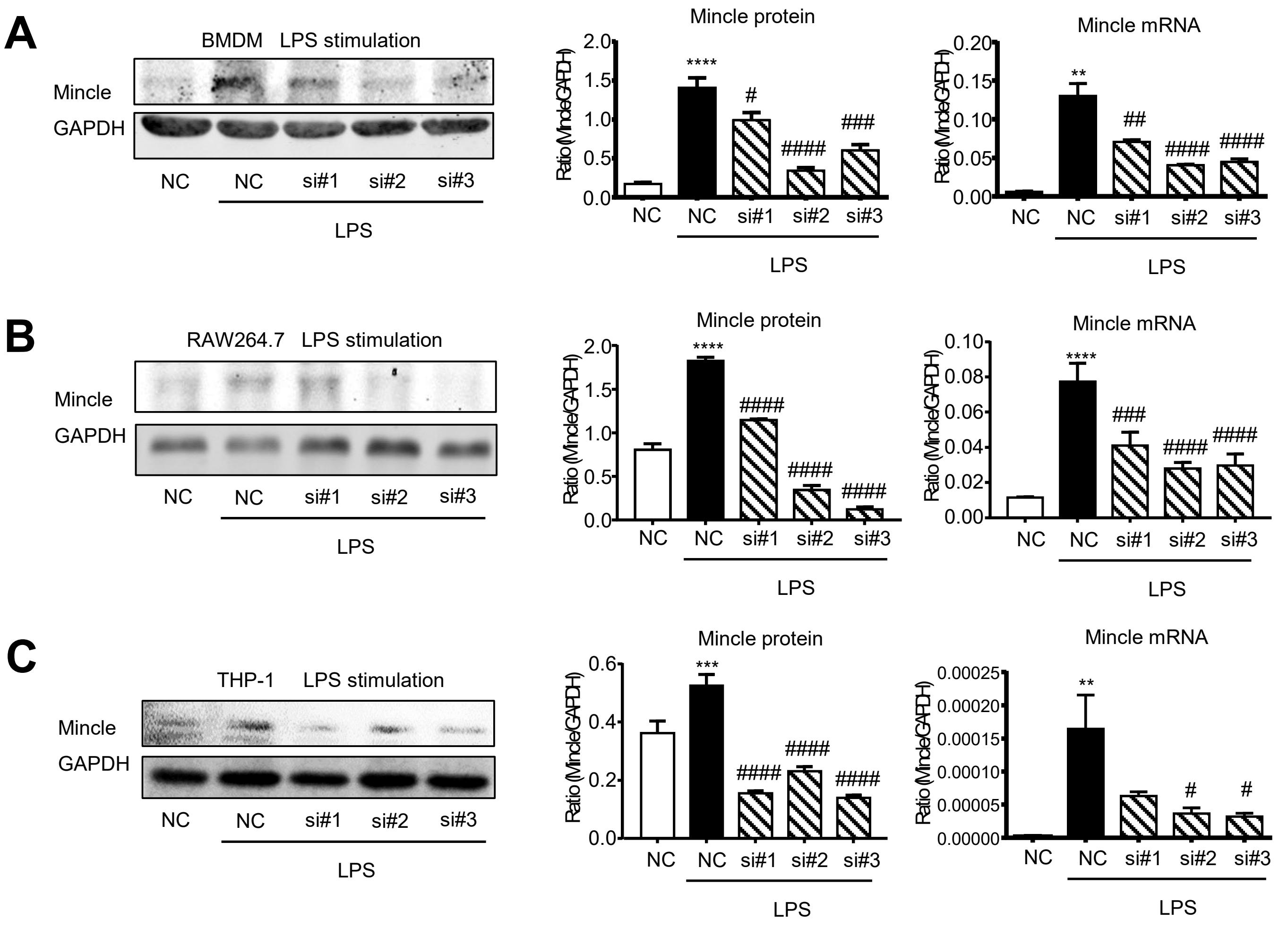

Figure S1. Screening of siRNA sequence for targeting both human and mouse Mincle *in vitro*. The knockdown efficiency of 3 siRNA sequences were tested on both murine and human macrophages in vitro. The siMincle #3 showed the best effect on Mincle suppression in mouse **(A)** BMDM and **(B)** RAW264.7 cells as well as human **(C)** THP-1 derived macrophages under LPS stimulation at 24h showing by Western blot and their quantification. Data represent mean ± SEM of 3 independent in vitro experiments. Statistical analysis based on ordinary one-way ANOVA, **** $p < 0.0001$, *** $p < 0.001$, ** $p < 0.01$ vs. NC; #### $p < 0.0001$, ### $p < 0.001$, ## $p < 0.01$, # $p < 0.05$ vs. NC+LPS.

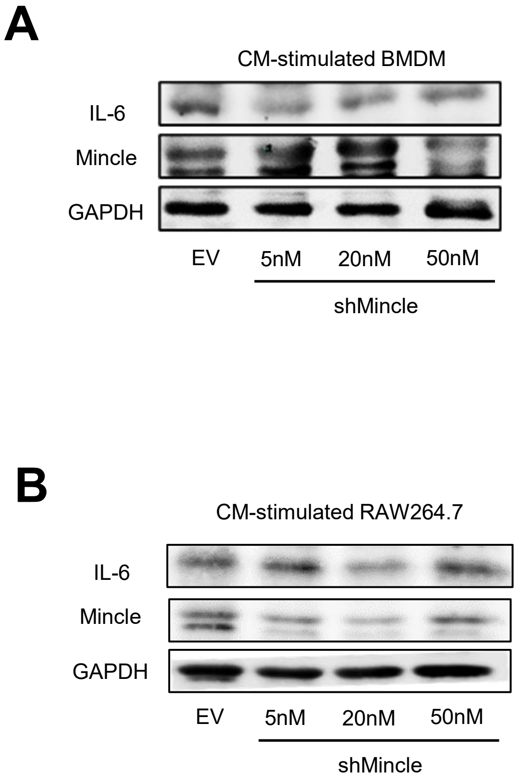

Figure S2. The shMincle effectively inhibits Mincle signaling *in vitro*. Western analysis confirmed that shMincle effectively inhibited the expression of Mincle and downstream effector IL-6 in **(A)** BMDM and **(B)** RAW264.7 cells under CM stimulation at 24h in vitro.

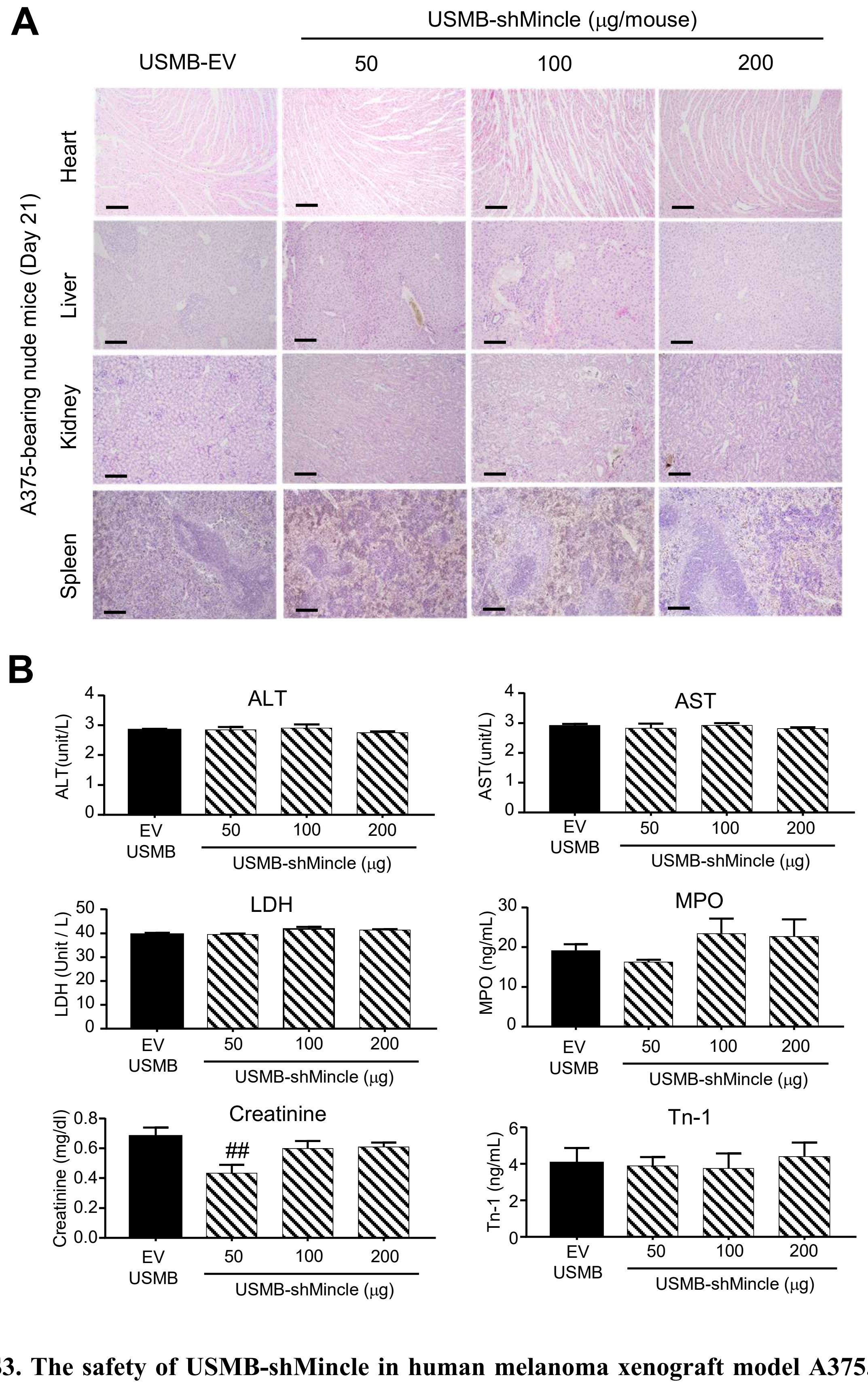

Figure S3. The safety of USMB-shMincle in human melanoma xenograft model A375. (A) H&E staining of important organs and **(B)** ELISA of the serum levels of ALT, AST, LDH, MPO, creatinine, and Tn-1 from the A375-bearing mice detected. Data represent mean ± SEM of 5 mice/group.

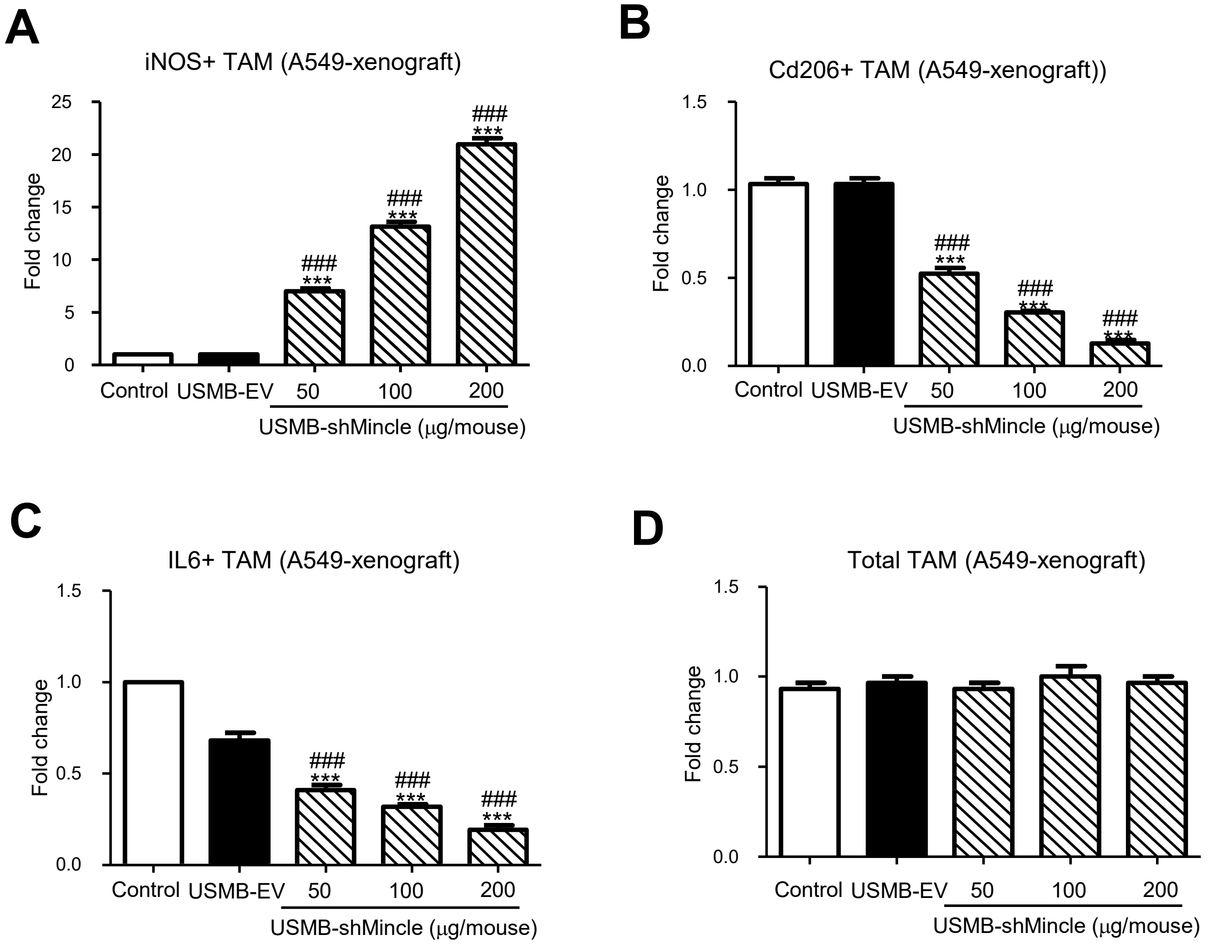

Figure S4. USMB-shMincle significantly altered the phenotype and function but not the number of TAM in the A549 xenografts *in vivo*. (A, B) The phenotypes of M1 (iNOS) and M2 (Cd206) and **(C)** expression of protumoral effector IL6 were dose-dependently changed in the USMB-shMincle compared to the baseline macrophage level of untreated control group, representing by the fold-change vs control group. **(D)** Interestingly, no significant change was detected in the number of TAMs among all groups by quantifying immunofluorescence result of Figure 6A. Data represent mean ± SEM of 5 mice/group. Statistical analysis based on ordinary one-way ANOVA.

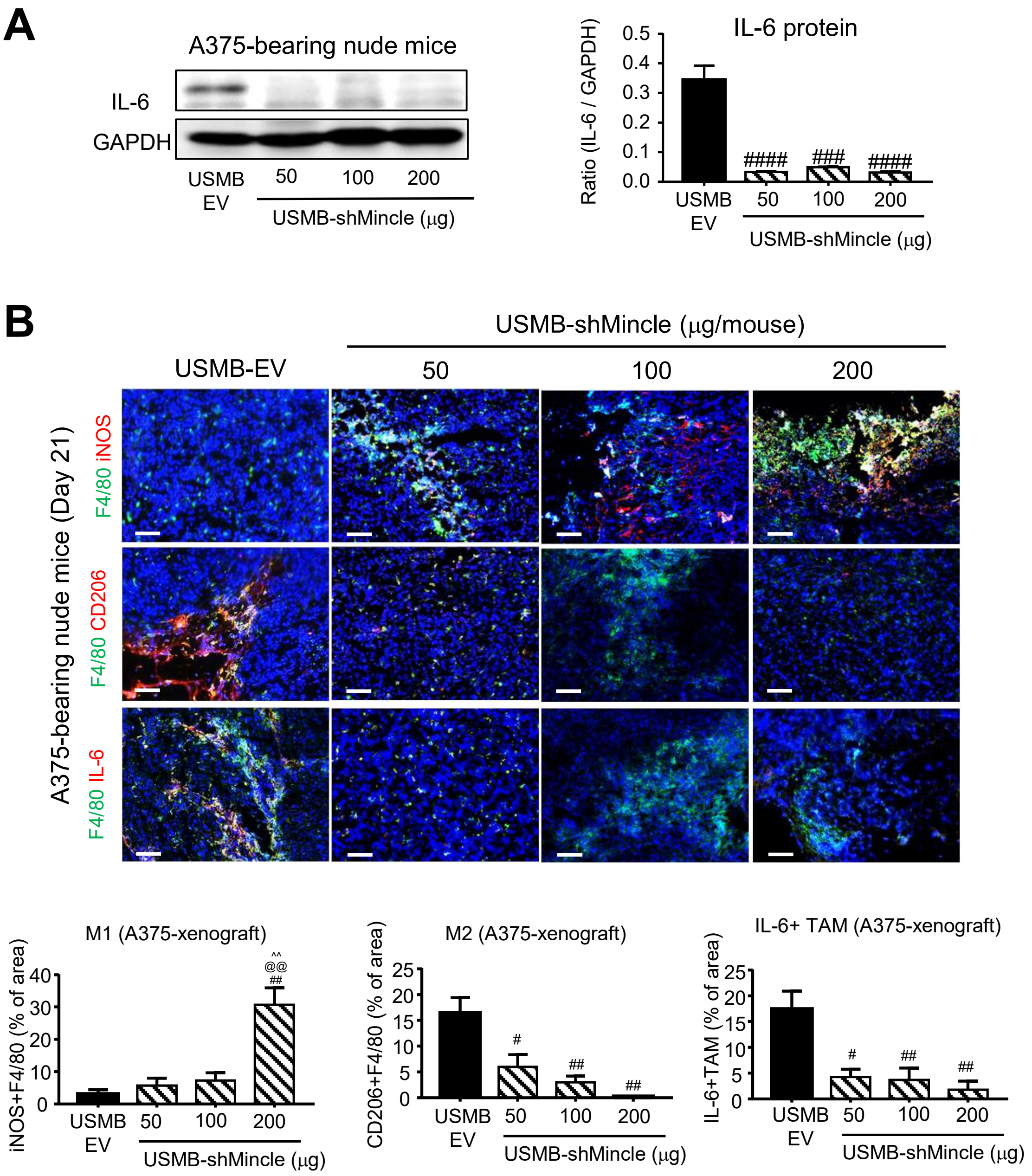

Figure S5. USMB-shMincle markedly inhibits M1/M2 transition in A375-bearing mice. **(A)** Treatment with USMB-shMincle markedly repressed the production of IL-6 in A375-xenografts showing by Western blot. **(B)** The USMB-shMincle largely enhanced the M1 phenotype iNOS but dramatically reduced the M2 phenotype CD206 and protumoral effector IL-6 in TAMs *in vivo*. Data represent mean ± SEM of 5 mice/group. Statistical analysis based on ordinary one-way ANOVA, #### $p < 0.0001$, ### $p < 0.001$, ## $p < 0.01$, # $p < 0.05$ vs. EV; @@ $p < 0.01$ vs. 50μg USMB-shMincle; ^^ $p < 0.01$ vs. 100μg USMB-shMincle.

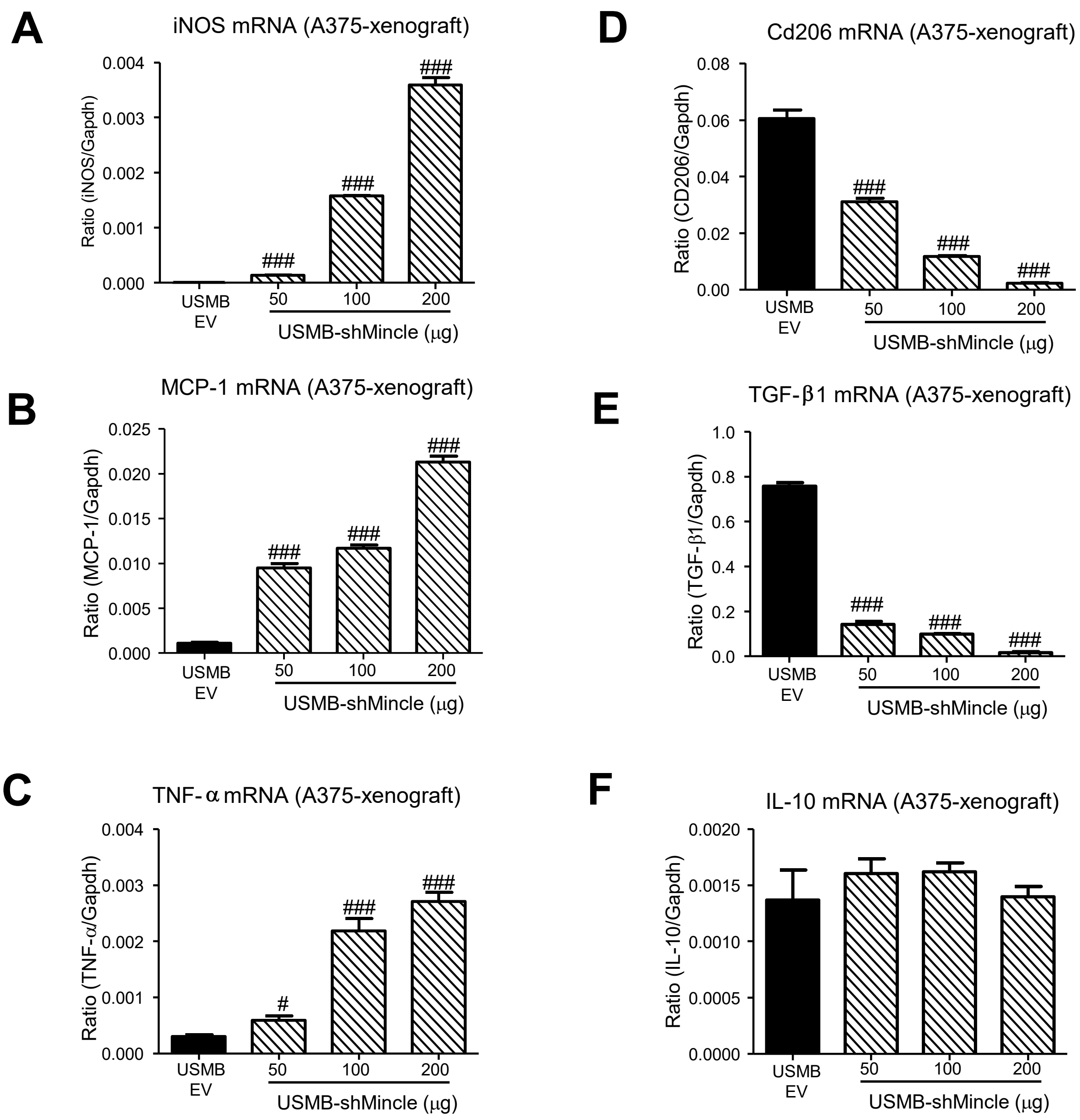

Figure S6. USMB-shMincle effectively enhances M1 function in A375-xenograft *in vivo*. Real-time PCR analysis showed that USMB-shMincle dose-dependently enhanced expressions of marker and effectors of **(A-C)** M1 (iNOS, MCP-1 and TNF-α), **(D-F)** but relatively reduction or insignificant change of M2 (Cd206, TGF-β1, IL-10) in the treated A375-xenografts. Data represent mean ± SEM of 5 mice/group. Statistical analysis based on ordinary one-way ANOVA, ### $p < 0.001$, # $p < 0.05$ vs. empty vector control (USMB-EV).

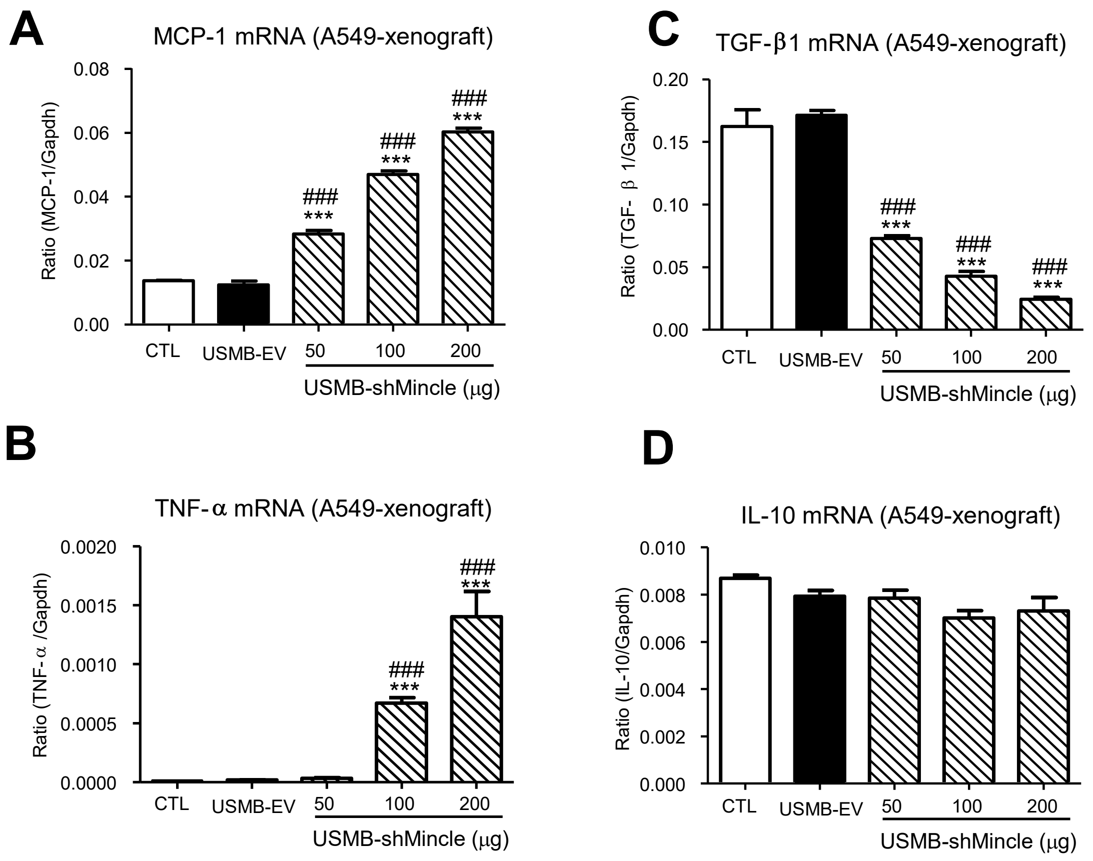

Figure S7. USMB-shMincle effectively enhances M1 function in A549-xenograft *in vivo*. Real-time PCR analysis showed that USMB-shMincle dose-dependently enhanced expressions of **(A-B)** M1 effectors MCP-1, TNF-α, **(C-D)** but relatively mild reduction or insignificant change of M2 effectors TGF-β1, IL-10 in the treated A549-xenografts in vivo. Data represent mean ± SEM of 5 mice/group. Statistical analysis based on ordinary one-way ANOVA, *** $p < 0.001$ vs. untreated control (CTL); ### $p < 0.001$ vs. empty vector control (USMB-EV).

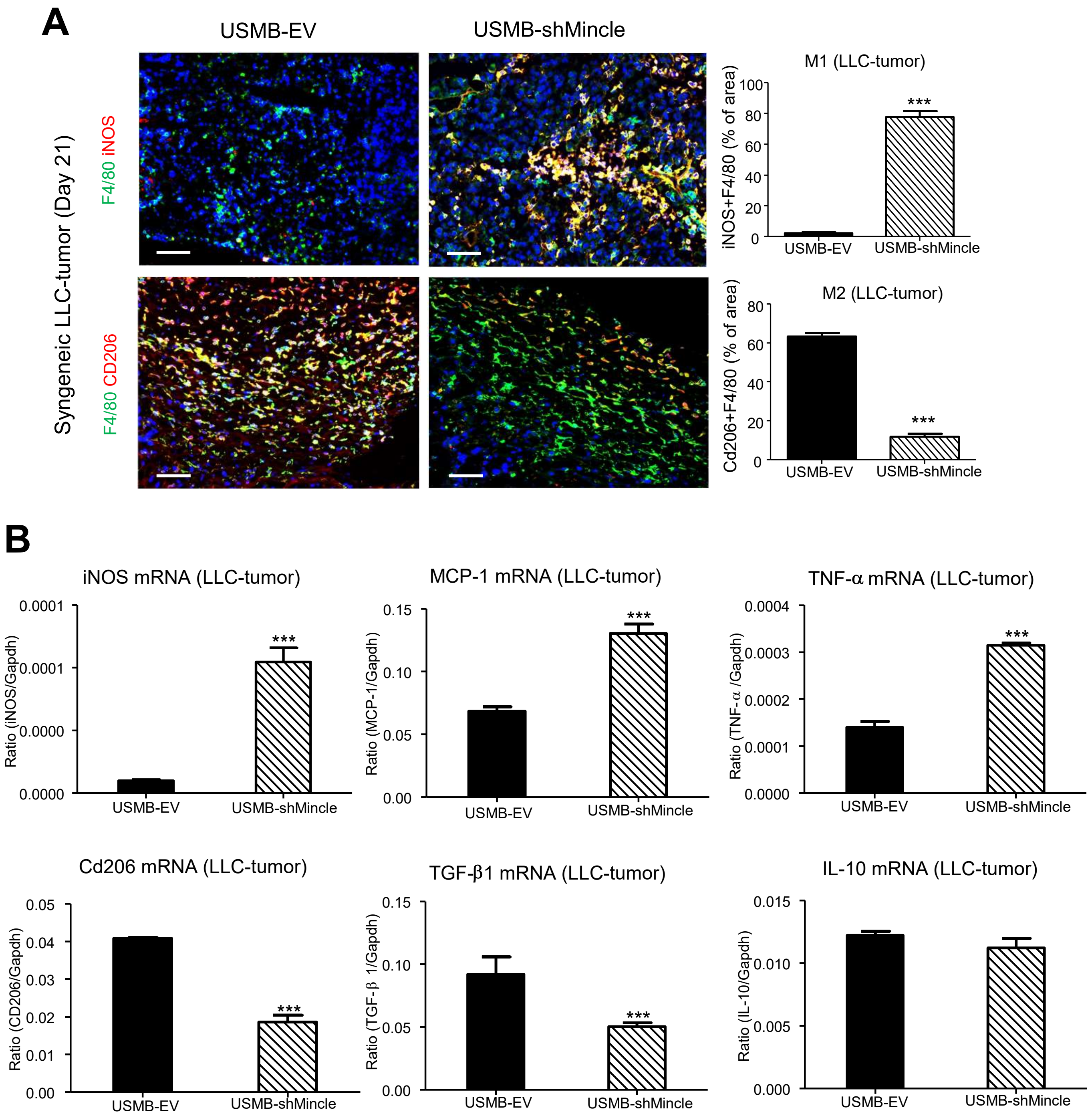

Figure S8. Effects of USMB-shMincle on TAM phenotypes and functions in syngeneic mouse lung carcinoma model LLC in vivo. (A) Immunofluorescence and **(B)** real-time PCR detects a significant increments of M1 (iNOS, MCP-1, TNF-α) but mild reduction or insignificant change of M2 (CD206, TGF-β1, IL-10) phenotypes and effectors in the LLC-tumor of immunocompetent C57BL/6J mice received USMB-shMincle compared to the mice received USMB-EV *in vivo*. Data represent mean ± SEM of 5 mice/group. *** $p < 0.001$ vs USMB-EV. Scale bars, 50 μm.

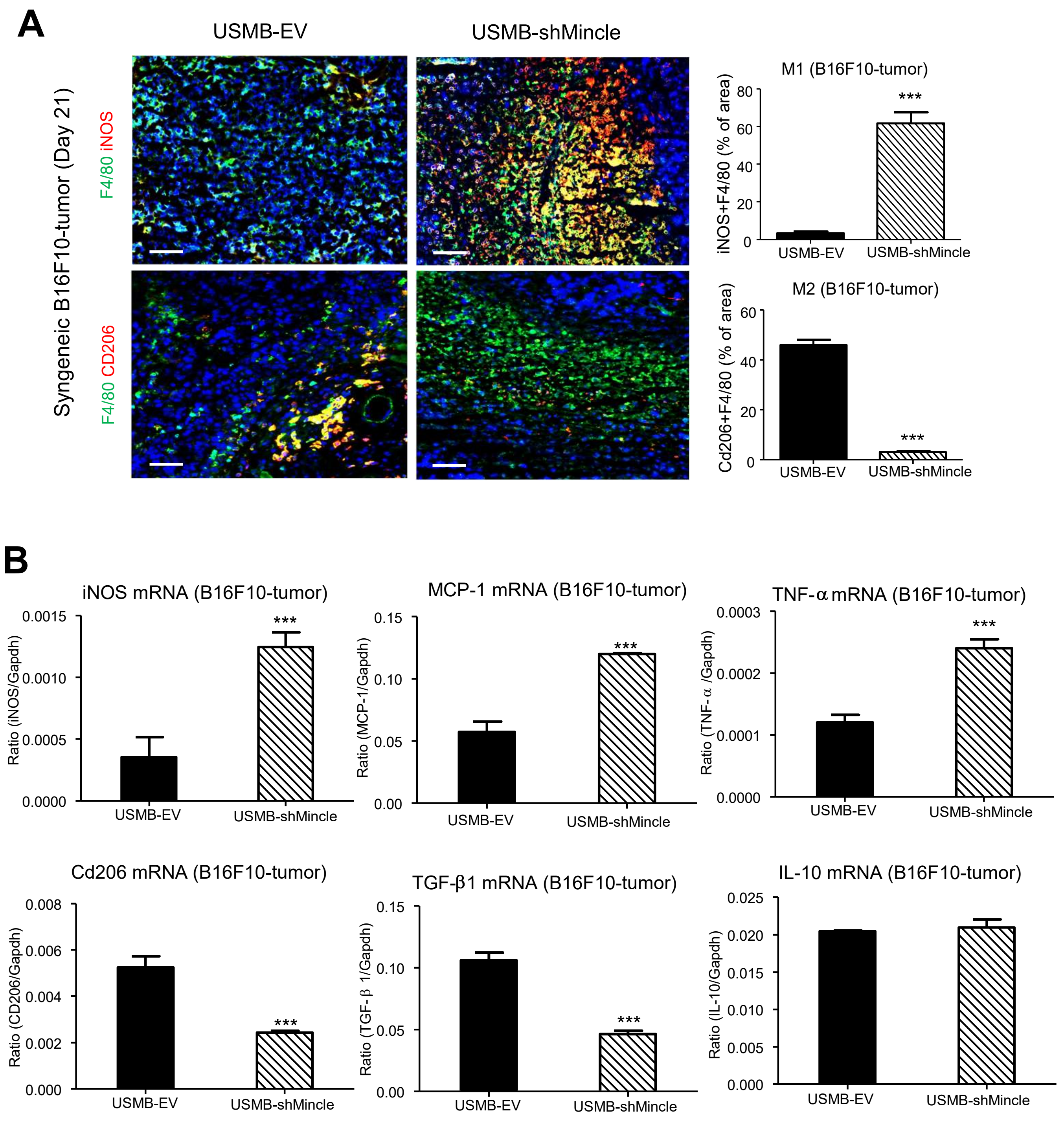

Figure S9. Effects of USMB-shMincle on TAM phenotypes and functions in syngeneic mouse melanoma model B16F10 in vivo. (A) Immunofluorescence and **(B)** real-time PCR detects a significant increments of M1 (iNOS, MCP-1, TNF-α) but mild reduction or insignificant change of M2 (CD206, TGF-β1, IL-10) phenotypes and effectors in the B16F10-tumor of immunocompetent C57BL/6J mice received USMB-shMincle compared to the mice received USMB-EV *in vivo*. Data represent mean ± SEM of 5 mice/group. *** $p < 0.001$ vs USMB-EV. Scale bars, 50 μm.

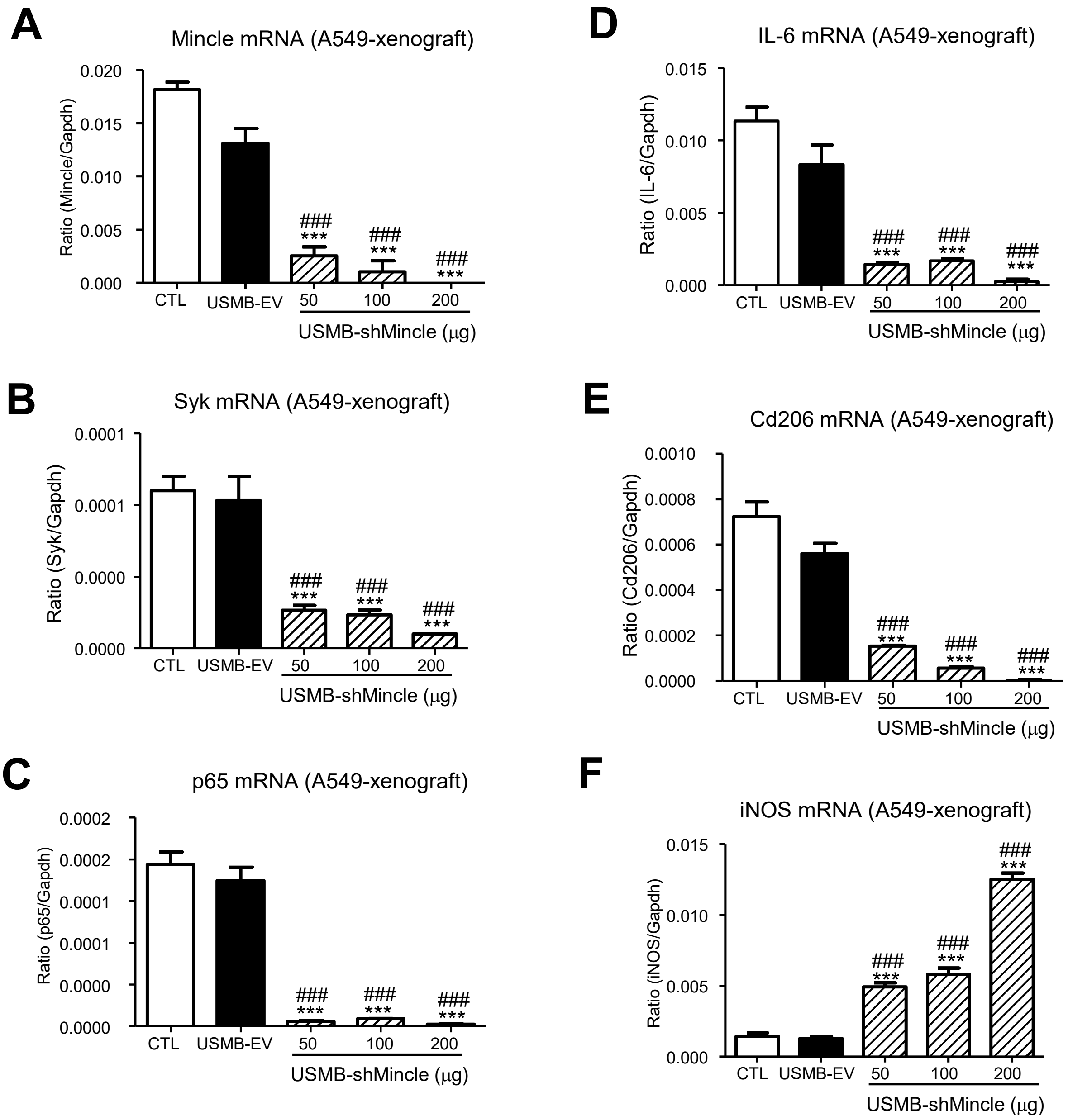

Figure S10. USMB-shMincle effectively blocks Mincle/Syk/NF-kB circuit and M1/M2 transition in A549-xenografts in vivo. Real-time PCR analysis showed that USMB-shMincle dose-dependently inhibits **(A-C)** Mincle/Syk/NF-kB circuit, **(D)** its protumoral effector IL-6, and **(E)** M2 phenotype Cd206 in the treated human NSCLC A549-xenografts. **(F)** Additionally, USMB-shMincle largely enhanced the M1 phenotype in the treated mice *in vivo*. Data represent mean ± SEM of 5 mice/group. Statistical analysis based on ordinary one-way ANOVA, *** $p < 0.001$ vs. CTL, ### $p < 0.001$ vs. EV.
